# A novel method for correcting scanline-observational bias of discontinuity orientation

**DOI:** 10.1038/srep22942

**Published:** 2016-03-10

**Authors:** Lei Huang, Huiming Tang, Qinwen Tan, Dingjian Wang, Liangqing Wang, Mutasim A. M. Ez Eldin, Changdong Li, Qiong Wu

**Affiliations:** 1 Faculty of Engineering, China University of Geosciences, Wuhan 430074, China; 2 Three Gorges Research Centre for Geo-hazard, Ministry of Education, Wuhan 430074, China; 3 College of Petroleum Geology & Minerals, University of Bahri, Khartoum 13314, Sudan

## Abstract

Scanline observation is known to introduce an angular bias into the probability distribution of orientation in three-dimensional space. In this paper, numerical solutions expressing the functional relationship between the scanline-observational distribution (in one-dimensional space) and the inherent distribution (in three-dimensional space) are derived using probability theory and calculus under the independence hypothesis of dip direction and dip angle. Based on these solutions, a novel method for obtaining the inherent distribution (also for correcting the bias) is proposed, an approach which includes two procedures: 1) Correcting the cumulative probabilities of orientation according to the solutions, and 2) Determining the distribution of the corrected orientations using approximation methods such as the one-sample Kolmogorov-Smirnov test. The inherent distribution corrected by the proposed method can be used for discrete fracture network (DFN) modelling, which is applied to such areas as rockmass stability evaluation, rockmass permeability analysis, rockmass quality calculation and other related fields. To maximize the correction capacity of the proposed method, the observed sample size is suggested through effectiveness tests for different distribution types, dispersions and sample sizes. The performance of the proposed method and the comparison of its correction capacity with existing methods are illustrated with two case studies.

Rockmass is a discrete medium composed of rock material and discontinuities including faults, fractures, joints, veins, bedding planes, cleavage planes, and schistosity planes, among others. Such discontinuities dominate the kinematical and mechanical behaviour of engineering rockmass^1^,^2^,^3^,^4^,^5^, with the analysis of this behaviour extending to various applications in such areas as rockmass stability evaluation, rockmass permeability analysis, rockmass quality calculation and other related fields, using three-dimensional rockmass models frequently generated via discrete fracture network (DFN) modelling with input geometrical variables including orientation^6^,^7^,^8^,^9^,^10^,^11^,^12^,^13^,^14^,^15^. These orientations are primarily measured on fresh rock exposures, with previous studies reporting various representative techniques^16^,^17^,^18^,^19^. More recently, a circular window sampling has been used to measure geometrical features^20^, and a careful window mapping was used for geometric fracture measurement at a surface outcrop in Kilve on the southern margin of the Bristol Channel Basin^21^, while a Lidar scanning technology was applied to determine the discontinuity orientation along a highway in Canada^22^,^23^.

However, some studies have used a line sampling technique, the most common being scanline sampling or borehole sampling. For example, scanlines were fixed on the rock faces of the San Manual copper mine in Arizona, USA, to collect orientations^24^. Boreholes were also used to obtain the orientation sample of a trending anticline at an anonymous field^25^. Both a single scanline and a multiple scanline method were used at Big Quarry site in northeast Wisconsin on the Door Peninsular between Lake Michigan and Green Bay^26^. These studies have shown that line sampling introduces a bias because a scanline preferably samples those discontinuities having large intersection angles, meaning the sampled probability increases with the intersection angle. To illustrate this bias, Supplementary Fig. S1(a) arbitrarily supposes two sets of discontinuities, where (1) the intensities of the two discontinuity sets are equal and (2) the intersection angle between the scanline and the Discontinuity Set 1 is smaller than that between the scanline and Set 2, that is, *θ*_1_ < *θ*_2_. Consequently, fewer discontinuities in Set 1 are sampled by the scanline than for Set 2. In the extreme case, none of Set 1 is sampled if *θ*_1_ = 0°, and consequently no orientation is observed in this set (Supplementary Fig. S1(b)). As this example suggests, it is easy to see that this angular bias exists in orientation observation using scanline, especially with small intersection angles.

The first approach for correcting this bias, the Terzaghi method which appeared in 1965, obtained the corrected frequency by dividing the observed frequency by the sine of intersection angle^27^. A detailed explanation of this method can be found in past research^28^. Since its introduction, most orientation corrections have adopted this method or one of its improved versions. For example, this method was applied to discontinuity orientation data obtained from scanline and borehole sampling in road cuts^29^. Subsequently, the application of this method was extended to curved scanlines and boreholes^30^, with a modification being applied to the sampling of fractures with a borehole and the sampling with the surface of a borehole^31^. A revision of this method, the Fouché method, which adds the discontinuity sample size into the original equation was developed to improve correction capacity^32^. The work reported here found that the correction of the Fouché method still results in a considerable error as mentioned in the Results Section. To address this issue, this paper proposes a more effective correction method. Firstly, the solutions for expressing the functional relationship between the distribution observed by a scanline (in one-dimensional space) and the inherent distribution (in three-dimensional space) are derived using probability theory and calculus (shown in the Supplementary Information). Secondly, based on these solutions, a novel method for correcting the bias is proposed, one which includes a hypothesis and two procedures (shown in Methods). Thirdly, the effect of the observed sample size on the correction capacity of the proposed method is examined based on 84 artificial datasets. Then, the optimal sample size, defined as that which can achieve the maximum correction capacity with few observations, is determined (shown in Results). Finally, the correction capacity of the proposed method is compared with the existing Fouché method using two discontinuity orientation samples, one from a lithic arkose exposure at Wenchuan, Sichuan, and a second from a dacite tunnel wall in Mankang, Tibet, China (shown in Results).

## Results

### The effect of sample size on correction capacity

When preparing a discontinuity survey, it is important to know the sample size required for maximizing the capacity of the bias correction, referred to as the optimal sample size. To analyse the effect of sample size on the capacity and to select the optimal sample size for the proposed method, we compared the accuracies of the correction results of 84 artificial datasets from 4 distribution types, 3 dispersions and 7 sample sizes.

The accuracy is expressed by the root square error between the true and the corrected probability densities. For dip direction this value is represented by


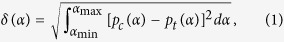


where *p*_*c*_(*α*) is the corrected probability density function of the dip direction, *p*_*t*_(*α*) the true probability density function of the dip direction, *α*_min_ the lower limit of the definition domain, *α*_max_ the upper limit of the definition domain, and *δ*(*α*) the root square error between the true and the corrected probability densities of the dip direction. For dip angle the root square error is calculated using the formula below,


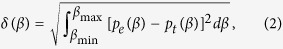


where *p*_*c*_(*β*) is the corrected probability density function of the dip angle, *p*_*t*_(*β*) the true probability density function of the dip angle, *β*_min_ the lower limit of the definition domain, *β*_max_ the upper limit of the definition domain, and *δ*(*β*) the root square error between the true and the corrected probability densities of the dip angle. The lower the root square errors *δ*(*α*) and *δ*(*β*), the more accurate the correction is.

**Figure 1:**
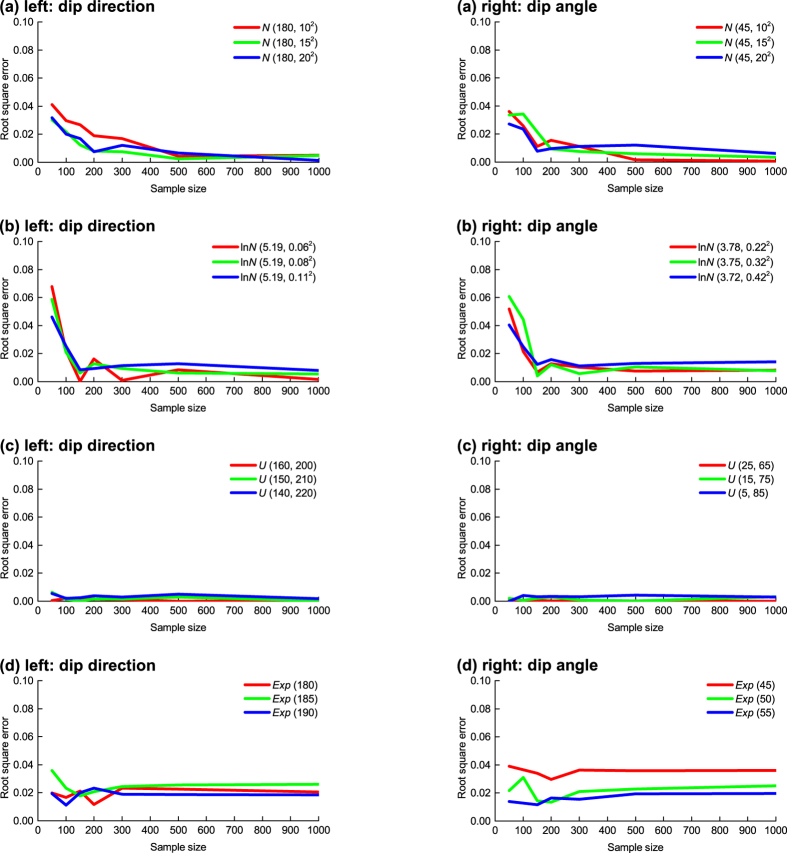
Root square error versus sample size curves: (**a**) Orientation follows normal distribution. Left is dip direction and right is dip angle. (**b**) Lognormal distribution. (**c**) Uniform distribution. (**d**) Exponential distribution.

The investigation method is shown in the Methods Section. The result (Fig. 1) shows:

- Normal distribution: The error curve of *N* (180, 10^2^) dip direction decreases from 4.1 × 10^−2^ to 4.4 × 10^−3^ with an average descent rate of 8.1 × 10^−5^ per sample size in the sample size interval of 50 to 500. For *N* (45, 10^2^) dip angle, the error curve decreases from 3.6 × 10^−2^ to 1.5 × 10^−3^ with an average descent rate of 7.7 × 10^−5^ per sample size in the same interval. In the adjacent interval over a sample size of 500, the curve for dip direction is approximately horizontal at 5.0 × 10^−3^ with an average ascendant rate of 1.2 × 10^−6^ per sample size; and the curve for dip angle is approximately horizontal at 7.3 × 10^−4^ with an average descent rate of 1.5 × 10^−6^ per sample size. This result reveals that the increase in sample size can improve correction capacity up to a sample size of 500. Similar trends can be found in the cases with more disperse dip directions/angles *N* (180, 15^2^)/*N* (45, 15^2^) and *N* (180, 20^2^)/*N* (45, 20^2^) although there exists a local minimum point at a sample size of 200 for *N* (180, 20^2^). So the sample size should be constrained below 500 for normally distributed orientations.
- Lognormal distribution: The error curve of ln*N* (5.19, 0.06^2^) dip direction decreases approximately linearly from 6.8 × 10^−2^ to 3.1 × 10^−4^ with an average descent rate of 6.7 × 10^−4^ per sample size in the sample size interval ranging from 50 to 150. For ln*N* (3.78, 022^2^) dip angle, the error curve decreases approximately linearly from 5.2 × 10^−2^ to 6.9 × 10^−3^ with an average descent rate of 4.5 × 10^−4^ per sample size in the same interval. In the adjacent interval over sample size 150, the error fluctuates and ultimately becomes stable. Similar overall trends are exhibited in the more disperse data of dip directions/angles ln*N* (5.19, 0.08^2^)/ln*N* (3.75, 0.32^2^) and ln*N* (5.19, 0.11^2^)/ln*N* (3.72, 0.42^2^), meaning that increasing the sample size can greatly improve the correction capacity up to a sample size of 150.
- Uniform distribution: The error curves of *U* (160, 200) dip direction/*U* (25, 65) dip angle are almost horizontal at 1.0 × 10^−3^/1.0 × 10^−4^. Similarly, in the cases of *U* (150, 210)/*U* (15, 75) and *U* (140, 220)/*U* (5, 85), the error curves are approximately horizontal at 2.9 × 10^−3^/2.0 × 10^−3^ and 2.1 × 10^−3^/3.2 × 10^−3^. These results suggest that the change in sample size has little effect on the correction capacity. Furthermore, these errors are close to 0, suggesting that the proposed method is quite effective for uniformly distributed orientations.
- Exponential distribution: The error curves of *Exp* (180) dip direction/*Exp* (45) dip angle reach up to more than 1.1 × 10^−2^/2.9 × 10^−2^. In particular, the error is more than 2.1 × 10^−2^ and 3.6 × 10^−2^ when the sample size is between 300 and 1000. Similarly, in the cases of *Exp* (185)/*Exp* (50) and *Exp* (190)/*Exp* (55), the errors are fairly large, more than 0.0177/0.0134 and 0.0111/0.0116. This shows the proposed method is unsuitable for exponentially distributed orientations.

The actual distribution type and dispersion of orientations of any of the samples are not known until the correction is calculated. Thus, taking into account these findings, the optimal sample size, if possible, is 150 for the proposed correction method. However, this optimal sample size is applicable for each discontinuity set rather than for the entire sample of multiple discontinuity sets, and it may vary depending on the correction method. In other words, the optimal sample size may be a different value or may not exist for various correction methods.

### Case I

This case of natural discontinuities is used to compare the correction capacities of the proposed and the existing methods. The existing methods for correcting orientation bias include the Terzaghi method^17^,^18^,^27^,^28^,^29^ and its modified versions^31^,^32^. The Terzaghi method obtains the corrected frequency by weighting the observed frequencies using the bias-compensatory factor:


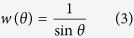


where *w* is the bias-compensatory factor, *θ* the intersection angle between the scanline and the discontinuity defined at each cell centre.

A modification of this method, the Fouché method, adds the discontinuity sample size to the original Terzaghi equation to improve the correction capacity^32^:


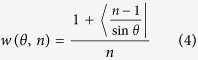


where *n* is the sample size of observed discontinuities and 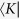 denotes the largest integer less than or equal to *K*. It was found that the original Terzaghi method is more applicable to the case *n* → ∞ while this modified method is applicable to an arbitrary value of *n*. The comparison conducted here is limited to the Fouché method.

Case I involves joints. The background and test method for Case I are shown in the Methods Section. The test returned two-tailed significances corresponding to the Fouché method of 0.739 for the dip direction and 0.782 for the dip angle, and for the proposed method of 0.913 for the dip direction and 0.918 for the dip angle. The significances for the proposed method are higher than that for the Fouché method, indicating that the proposed method performs more effectively than the Fouché method.

### Case II

**Figure 2:**
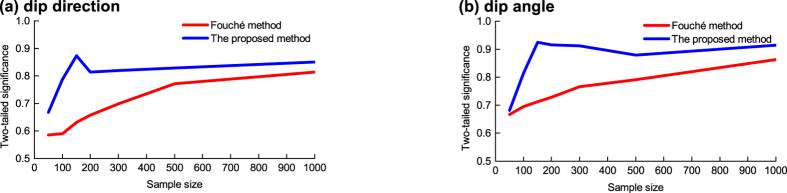
Two-tailed significance returned by the two-sample Kolmogorov-Smirnov test (Case II). This significance is used to quantify the distribution difference between the observed and the “modelled” orientations. The orientation is comprised of two components: (**a**) Dip direction. (**b**) Dip angle.

To support the comparison result above, a case with natural discontinuities, Case II, is used. In addition, this case is further used to check the optimal sample size previously determined from the artificial data. Case II involves bedding planes. The background and test method for this case are shown in the Methods Section. The result (Fig. 2) indicates that for the proposed method, the highest correction capacity is achieved at a sample size of approximately 150; when the sample size is over 150, the correction capacity does not significantly improve as the sample size increases. This result is in good agreement with the optimal sample size previously determined from the artificial data. Additionally, the comparison of significances corresponding to the Fouché method and the proposed method in this figure reveals that the proposed method provides a more accurate correction result than the Fouché method.

## Discussion

As suggested in the Results Section, the method proposed here is more effective than the Fouché method because of its higher correction capacity. The reason for this is that the Terzaghi method is based on the assumption that all of the discontinuities in each counting cell are parallel^33^ (see Supplementary Appendix A). Because all discontinuities are not parallel, this discrepancy between the assumption and the fact leads to an error. The revised version of this method is also based on this assumption, meaning the Fouché method includes the same error. Unlike the Fouché method, the proposed method does not make this assumption and, thus, does not introduce this error.

The proposed method is derived from Supplementary Equation (11) based on an implicit assumption that the sampling tool is 0 m in diameter, an assumption fulfilled by a scanline. However, it is not clear if this method can be applied to diameters greater than 0 m, for example a borehole/well. We believe that the applicability of this proposed method in this situation could be determined if adequate fracture data from boreholes can be obtained, or more importantly, a revised method could be derived as long as the underlying Supplementary Equation (11) can be rewritten to address a diameter greater than 0 m. While this study did not include these data, future research can investigate the borehole/well case.

The proposed method is based on probability theory, which assumes that discontinuity orientations follow probability distributions, an assumption also made by numerous geologists. More specifically, many geologists have assumed or verified that the orientations of fractures investigated by the line sampling technique follow several theoretical random distribution types, such as the uniform distribution^34^,^35^, exponential distribution^36^, normal distribution^37^,^38^,^39^,^40^, Fisher distribution^41^,^42^,^43^,^44^, Kent distribution^45^,^46^,^47^, Weibull distribution^48^, and Bingham distribution^49^,^50^,^51^,^52^,^53^. In addition, engineers who have found that these theoretical distribution types do not fit the orientation sample have concluded that the data may follow empirical probability distributions not yet reported^54^,^55^; some observed orientation samples may fit a lognormal distribution. Hence, the probability distribution assumption is reasonable for most observed fractures. Unlike fractures, another type of discontinuity, bedding planes, are nearly parallel. In fact, the orientation of bedding planes can be considered as a special case following a probability distribution defined within a narrow domain, for instance from 132 to 141° in dip direction and from 68 to 76° in dip angle shown here in Case I. From this perspective, such a special case of sub-parallel discontinuities, thus, belongs to a probability distribution, where the proposed method can be applied.

**Figure 3:**
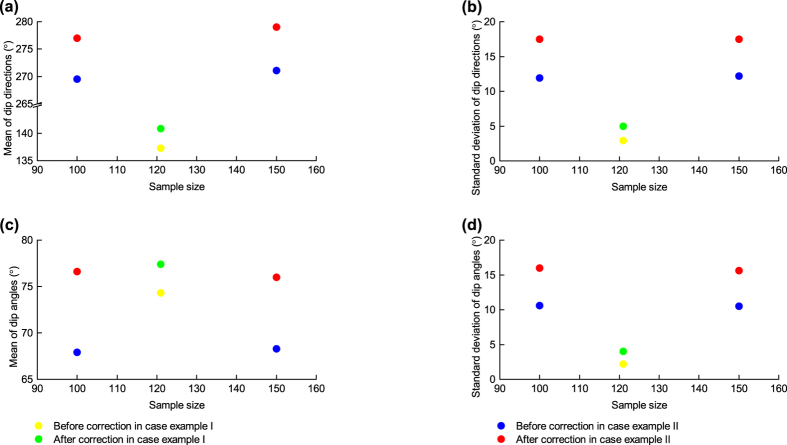
Orientation distribution parameters before and after correction using the proposed method: (**a**) Mean of dip directions. (**b**) Standard deviation of dip directions. (**c**) Mean of dip angles. (**d**) Standard deviation of dip angles.

The distribution after correction in Case I is altered slightly relative to the raw distribution, while that in Case II is altered more significantly as seen in Fig. 3 even though the averages of the intersection angles between the scanline and the discontinuities (48.1° for Case I and 51.7° for Case II) are similar. The dispersion of the orientations of the non-parallel discontinuities (e.g. 12.1° and 10.0° standard deviations corresponding to dip directions and dip angles, respectively, of the 1,016 joints in Case II) is responsible for this observational bias. Compared with joints, sub-parallel discontinuities are less dispersed in orientation (e.g. 2.9° and 2.2° standard deviations corresponding to dip directions and angles of bedding planes, respectively, in Case I), meaning that the observational bias is smaller, even negligible at times. Consequently, the correction for bedding planes seems less significant than for joints. In particular, for extremely parallel bedding planes, the correction may not be required due to two primary reasons. The first is that, to our knowledge, the observational bias is so small that the data sufficiently approximate the distribution in three-dimensional space which is just required for DFN modelling. The second is that the improved accuracy will be very limited even using the proposed method, owing to the appropriately high approximation of the raw data in relation to the three-dimensional distribution as mentioned previously. Our next study will analyse this issue more fully.

The proposed method uses dip direction/dip angle to delineate orientation. This linear delineation is limited because when a discontinuity cluster crosses the 0° dip direction, the statistics of dip direction will break down. However, the conversions of the dip direction in Supplementary Appendix B can be used to avoid this statistical break. A similar limitation may appear with a discontinuity cluster close to the 0° dip angle, where the same conversion of the dip direction is also required before correction.

In addition to the orientation focused on in this paper, another geometric element of discontinuity, volume intensity, sometimes referred to as volume density, is important in DFN modelling. Three aspects are needed to calculate volume intensity, scanline or borehole orientation, the orientation probability distribution, and the diameter or radius probability distribution. As a prerequisite for this calculation, the orientation probability distribution corrected by the proposed method may contribute to a more accurate intensity calculation.

## Conclusions

This paper presents a novel method for correcting the scanline-observational bias of discontinuity orientation. This method includes two steps that 1) correct the cumulative probabilities of orientation based on numerical solutions of orientation probability density in three-dimensional space and 2) determine the distribution of the corrected orientations using approximation methods. The numerical solutions in the first step were derived using probability theory and calculus under the distribution independence hypotheses of dip direction and dip angle. The approximation in the second step can be easily implemented using the one-sample Kolmogorov-Smirnov test. The results corrected by the proposed method, the probability distribution function of orientation in three-dimensional space, can be used as one of the parameters for discrete fracture network (DFN) modelling. As is known, DFN can be applied in various research areas, including the evaluation of rockmass stability, the analysis of rockmass permeability, the calculation of rockmass quality and other such related fields.

To maximize the correction capacity of the proposed method with few observations, the optimal sample size is determined from capacity comparisons of different sample sizes, distribution types and dispersions of orientation data. The results revealed that the highest correction capacity is achieved at an approximate sample size of 150; once it exceeds this value, the correction capacity does not significantly improve with a larger sample size, meaning the optimal sample size tends to be this value. However, this optimal sample size is only applicable for each discontinuity set, not the entire sample comprised of multiple discontinuity sets, and the determination of the optimal sample size depends on the correction method. In other words, the optimal sample size is approximately 150 for the proposed method, but may take different values or even not exist for other correction methods. This optimal sample size determined from artificial data is supported by an actual orientation sample of dacite joints exposed on a tunnel wall in Mankang, Tibet, China.

The proposed method was subsequently compared with the existing Fouché method using two observed orientation samples from both the tunnel wall and a second case involving lithic arkose beddings exposed on an outcrop in Wenchuan, Sichuan, China. The results demonstrate that the proposed method provides a more accurate corrected orientation distribution for DFN modelling than the Fouché method. The reasons for this higher correction capacity of the proposed method were discussed as well as the low correction capacity when applied to bedding planes.

## Methods

### The proposed method

The proposed method is based on numerical approximate solutions and includes one hypothesis and two procedures. Supplementary Appendix C presents a detailed derivation of the functional relationship between the observed distribution by scanline (in one-dimensional space) and the inherent distribution (in three-dimensional space). The derived result shows that it is difficult to obtain the exact analytic solutions of the inherent distribution because of an unsolved integral. Thus, numerical approximate solutions are derived in terms of piecewise functions (see Supplementary Equations (33) for dip direction and (34) for dip angle).

#### Hypothesis

As shown in Supplementary Appendix C, the numerical approximate solutions are derived under the hypothesis that the observed dip direction and dip angle are independent of each other. The proposed method uses these solutions and accordingly relies on this hypothesis. Although the dip direction and dip angle are dependent for some discontinuities like bedding planes in plunging folds^56^, there are several cases that consider them as independent variables^17^,^34^,^35^,^36^,^42^,^43^,^44^,^57^,^58^,^59^,^60^. For this reason, it is necessary to check whether the observed orientation sample meets the independence hypothesis before using the proposed method. Only if the independence test is met, can the proposed method be applied.

There are many methods for checking for independence, one of which is the Pearson’s chi-square (χ^2^) test. This method assesses whether paired observations of two variables are independent of each other as expressed in a contingency table. It returns a two-tailed significance characterizing the independence ranging from 0 to 1; the greater the value, the more significant is the independence. It is generally believed that the independence hypothesis can be accepted if the significance is above the confidence level of 0.05. Its properties were first investigated in the last century^61^ and further details can be found in recent literature^62^,^63^.

#### Procedure

The procedure for the proposed method is as follows.

1. Correct the cumulative probabilities of the dip direction and angle based on the numerical solutions Supplementary Equations (33) and (34), respectively.
2. Determine the distribution of the corrected orientations using approximation methods such as the one-sample Kolmogorov-Smirnov test. This nonparametric test was developed to compare a sample with a hypothesized probability distribution^64^,^65^. It returns a two-tailed significance quantifying the distance between the empirical distribution function of the sample and the cumulative distribution function of the hypothesized distribution. This test is usually applied to approximating the distribution of a sample with a hypothesized distribution type^66^,^67^. It allows for various hypothesized distribution types to be selected to obtain the fittest distribution.

### Investigation of the effect of sample size

This investigation focuses on the effect of sample size on the correction capacity of the proposed method. It contains the following four steps.

Firstly, the true distribution of the orientations is arbitrarily hypothesized, in addition to other geometric parameters that are necessary for the modelling as listed in Table 1. This hypothesized orientation includes 4 common probability distribution types: normal, lognormal, uniform and exponential. For each distribution type, dispersion is set at 3 different values, while for each dispersion, the sample size is assigned 7 different values: 50, 100, 150, 200, 300, 500 and 1,000.

Then, entering these parameters into discrete fracture modelling as described in the previous literature^68^,^69^,^70^ results in the construction of 12 models of discontinuity networks, corresponding to the 12 combinations of the 4 distribution types and the 3 dispersions. Supplementary Fig. S2 shows only the model for the uniform distribution (i.e., Groups 43–49 in Table 1) due to space constraints.

Thirdly, the observations for each of the 12 models are conducted under 7 different sample sizes, so that a total of 84 samples of orientations of discontinuities are derived. Supplementary Fig. S3 shows only the observed samples from Groups 7, 28, 49 and 70 in Table 1. A Pearson’s chi-square (χ^2^) test is then executed to check the independence of the observed dip directions and dip angles, with results listed in Supplementary Table S1. As this table shows, the two-tailed significances are all above the confidence level of 0.05, indicating that the observed orientations meet the independence hypothesis, meaning the proposed method can be used.

Finally, the 84 observation samples were corrected using the proposed method, with results being listed in Table 2. Then, the root square errors between the corrected and the true distributions are calculated based on Equations (1) and (2), with results being shown in Fig. 1.

#### Case I

**Figure 4:**
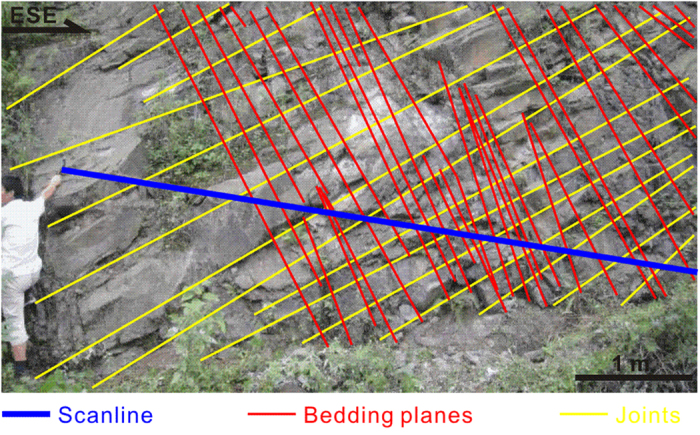
Field outcrop, scanline and discontinuities (Case I). The outcrop is the surface of a rock cut slope located near Yingxiu town in Wenchuan, Sichuan Province, China, about 1,800 m east of the epicentre of the 2008 Wenchuan earthquake and consists of Upper Triassic lithic arkose of the Xujiahe Formation. Two primary sets of discontinuities, i.e. bedding plane and joint, have developed in the rock.

The study area of this case is located near Yingxiu town in Wenchuan, Sichuan Province, China, about 1,800 m east of the epicentre of the 2008 Wenchuan earthquake. The specific roadcut is 11 m long, 5 m wide and 6 m high, and consists of Upper Triassic lithic arkose of the Xujiahe Formation. The rockmass has two primary discontinuity sets, one of which comprises the bedding planes seen in Fig. 4.

A scanline with the trend/plunge 108/15° was fixed on the outcrop to sample these bedding planes. Their orientations were measured with a geologic compass. As geologists have reported^71^,^72^,^73^,^74^,^75^, a single measurement by a geologic compass will introduce a large measurement error to the bedding plane orientation data. Because of this error, the data are frequently unable to represent the natural distribution. To reduce such an error, ten repeated measurements were conducted here and their average was considered as the observed orientation. Supplementary Fig. S4(a) shows the orientations of 121 observed bedding planes. A Pearson’s chi-square (χ^2^) test was used to calculate the independence of the dip direction and dip angle. The two-tailed significance obtained from this test was 0.86, above the confidence level of 0.05, indicating that the observed orientations met the independence hypothesis of the proposed method, meaning it can be used for these observed orientations.

Firstly, the sampling bias of the observed orientations was corrected using both the Fouché method and the proposed method. The result corrected by the Fouché method is shown in Supplementary Fig. S4(b); the result corrected using the proposed method indicated that the dip direction follows the normal distribution *N*(140.9, 5.0^2^) and the dip angle, the normal distribution *N*(77.4, 4.0^2^). Moreover, other essential parameters for modelling, specifically the volumetric intensity, the diameter and the aperture, were calculated, with the results being listed in Table 3.

Secondly, using these corrected geometric parameters, two three-dimensional models of the rock were constructed (see Supplementary Fig. S5). A scanline with the same orientation as the field scanline was applied to the model outcrop, and the discontinuities intersected by this scanline were then “measured”. Here, the sample size of these “measured” discontinuities was set equal to that of the observed discontinuities in the field. To distinguish them from the discontinuities observed, these “measured” discontinuities generated artificially were named “modelled” discontinuities. Supplementary Fig. S6 shows these “modelled” discontinuity orientations.

Finally, the distribution difference between the observed and the “modelled” orientations was tested using the two-sample Kolmogorov-Smirnov test, a nonparametric hypothesis test that evaluates the difference between the cumulative distribution functions of two sample data vectors^76^,^77^,^78^,^79^,^80^. This test returns a two-tailed significance characterizing the difference. The significance ranges from 0 to 1, with the higher the significance, the lower the difference. The result is shown in the text of the Results Section.

#### Case II

This case involves the Rumei Dam slope on the Lancang River, located in Mankang, Tibet, China (29° 34′ 30.0″ N, 98° 20′ 49.2″ E). The study area is primarily composed of two exposed strata: one is a light-gray, ash-black or dark-green dacite originating from the Zhuka Formation, Triassic, and the other is a fuchsia or lime-green sandstone from the Huakai Formation, Middle Jurassic. Three primary sets of discontinuities (bedding, Joint 1 and Joint 2) can be found in the rockmass. We conducted a geometrical measurement of the discontinuity orientation, spacing, aperture and trace length along a scanline with a trend/plunge of 243/4° on a tunnel wall. This paper considers only Joint 1; the orientations observed with a compass are shown in Supplementary Fig. S7(a). To check the independence of the dip direction and dip angle, a Pearson’s chi-square test was executed on the orientation sample, resulting in a significance of 0.56, above the confidence level of 0.05, suggesting that the observed orientation sample satisfies the independence hypothesis of the proposed method, meaning it can be applied to this sample.

Similar to the steps used in Case I, the orientations corrected using the Fouché method (see, for example, Supplementary Fig. S7(b) showing the corrected result from the sample of the first 1,000 observed orientations) and that using the proposed method (in terms of probability functions; see Table 4) were obtained, as well as the “modelled” orientations derived from the corrected results of these two methods (see Supplementary Fig. S7(c) and (d)). Then, the significances of 7 sample sizes (the first 50, 100, 150, 200, 300, 500 and 1,000 of 1,016 observed orientations) were tested, with the results being shown in Fig. 2.

## Additional Information

**How to cite this article**: Huang, L. *et al.* A novel method for correcting scanline-observational bias of discontinuity orientation. *Sci. Rep.* **6**, 22942; doi: 10.1038/srep22942 (2016).

## Supplementary Material

Supplementary Information

## Figures and Tables

**Table 1:** Parameters for discontinuity modelling.

Volumetric intensity (m^-3^)	Simulated zone			Applied zone			Scanline	
	Length (m)	Width (m)	Height (m)	Length (m)	Width (m)	Height (m)	Trend (°)	Plunge (°)
7	30	30	30	20	20	20	0	45
Group	True dip direction(°)		True dip angle (°)		Radius (m)		Sample size	
1	*N* (180, 10^2^)		*N* (45, 10^2^)		*Exp* (1.5)		50	
2	*N* (180, 10^2^)		*N* (45, 10^2^)		*Exp* (1.5)		100	
3	*N* (180, 10^2^)		*N* (45, 10^2^)		*Exp* (1.5)		150	
4	*N* (180, 10^2^)		*N* (45, 10^2^)		*Exp* (1.5)		200	
5	*N* (180, 10^2^)		*N* (45, 10^2^)		*Exp* (1.5)		300	
6	*N* (180, 10^2^)		*N* (45, 10^2^)		*Exp* (1.5)		500	
7	*N* (180, 10^2^)		*N* (45, 10^2^)		*Exp* (1.5)		1000	
8	*N* (180, 15^2^)		*N* (45, 15^2^)		*Exp* (1.5)		50	
9	*N* (180, 15^2^)		*N* (45, 15^2^)		*Exp* (1.5)		100	
10	*N* (180, 15^2^)		*N* (45, 15^2^)		*Exp* (1.5)		150	
11	*N* (180, 15^2^)		*N* (45, 15^2^)		*Exp* (1.5)		200	
12	*N* (180, 15^2^)		*N* (45, 15^2^)		*Exp* (1.5)		300	
13	*N* (180, 15^2^)		*N* (45, 15^2^)		*Exp* (1.5)		500	
14	*N* (180, 15^2^)		*N* (45, 15^2^)		*Exp* (1.5)		1000	
15	*N* (180, 20^2^)		*N* (45, 20^2^)		*Exp* (1.5)		50	
16	*N* (180, 20^2^)		*N* (45, 20^2^)		*Exp* (1.5)		100	
17	*N* (180, 20^2^)		*N* (45, 20^2^)		*Exp* (1.5)		150	
18	*N* (180, 20^2^)		*N* (45, 20^2^)		*Exp* (1.5)		200	
19	*N* (180, 20^2^)		*N* (45, 20^2^)		*Exp* (1.5)		300	
20	*N* (180, 20^2^)		*N* (45, 20^2^)		*Exp* (1.5)		500	
21	*N* (180, 20^2^)		*N* (45, 20^2^)		*Exp* (1.5)		1000	
22	ln*N* (5.19, 0.06^2^)		ln*N* (3.78, 0.22^2^)		*Exp* (1.5)		50	
23	ln*N* (5.19, 0.06^2^)		ln*N* (3.78, 0.22^2^)		*Exp* (1.5)		100	
24	ln*N* (5.19, 0.06^2^)		ln*N* (3.78, 0.22^2^)		*Exp* (1.5)		150	
25	ln*N* (5.19, 0.06^2^)		ln*N* (3.78, 0.22^2^)		*Exp* (1.5)		200	
26	ln*N* (5.19, 0.06^2^)		ln*N* (3.78, 0.22^2^)		*Exp* (1.5)		300	
27	ln*N* (5.19, 0.06^2^)		ln*N* (3.78, 0.22^2^)		*Exp* (1.5)		500	
28	ln*N* (5.19, 0.06^2^)		ln*N* (3.78, 0.22^2^)		*Exp* (1.5)		1000	
29	ln*N* (5.19, 0.08^2^)		ln*N* (3.75, 0.32^2^)		*Exp* (1.5)		50	
30	ln*N* (5.19, 0.08^2^)		ln*N* (3.75, 0.32^2^)		*Exp* (1.5)		100	
31	ln*N* (5.19, 0.08^2^)		ln*N* (3.75, 0.32^2^)		*Exp* (1.5)		150	
32	ln*N* (5.19, 0.08^2^)		ln*N* (3.75, 0.32^2^)		*Exp* (1.5)		200	
33	ln*N* (5.19, 0.08^2^)		ln*N* (3.75, 0.32^2^)		*Exp* (1.5)		300	
34	ln*N* (5.19, 0.08^2^)		ln*N* (3.75, 0.32^2^)		*Exp* (1.5)		500	
35	ln*N* (5.19, 0.08^2^)		ln*N* (3.75, 0.32^2^)		*Exp* (1.5)		1000	
36	ln*N* (5.19, 0.11^2^)		ln*N* (3.72, 0.42^2^)		*Exp* (1.5)		50	
37	ln*N* (5.19, 0.11^2^)		ln*N* (3.72, 0.42^2^)		*Exp* (1.5)		100	
38	ln*N* (5.19, 0.11^2^)		ln*N* (3.72, 0.42^2^)		*Exp* (1.5)		150	
39	ln*N* (5.19, 0.11^2^)		ln*N* (3.72, 0.42^2^)		*Exp* (1.5)		200	
40	ln*N* (5.19, 0.11^2^)		ln*N* (3.72, 0.42^2^)		*Exp* (1.5)		300	
41	ln*N* (5.19, 0.11^2^)		ln*N* (3.72, 0.42^2^)		*Exp* (1.5)		500	
42	ln*N* (5.19, 0.11^2^)		ln*N* (3.72, 0.42^2^)		*Exp* (1.5)		1000	
43	*U* (160, 200)		*U* (25, 65)		*Exp* (1.5)		50	
44	*U* (160, 200)		*U* (25, 65)		*Exp* (1.5)		100	
45	*U* (160, 200)		*U* (25, 65)		*Exp* (1.5)		150	
46	*U* (160, 200)		*U* (25, 65)		*Exp* (1.5)		200	
47	*U* (160, 200)		*U* (25, 65)		*Exp* (1.5)		300	
48	*U* (160, 200)		*U* (25, 65)		*Exp* (1.5)		500	
49	*U* (160, 200)		*U* (25, 65)		*Exp* (1.5)		1000	
50	*U* (150, 210)		*U* (15, 75)		*Exp* (1.5)		50	
51	*U* (150, 210)		*U* (15, 75)		*Exp* (1.5)		100	
52	*U* (150, 210)		*U* (15, 75)		*Exp* (1.5)		150	
53	*U* (150, 210)		*U* (15, 75)		*Exp* (1.5)		200	
54	*U* (150, 210)		*U* (15, 75)		*Exp* (1.5)		300	
55	*U* (150, 210)		*U* (15, 75)		*Exp* (1.5)		500	
56	*U* (150, 210)		*U* (15, 75)		*Exp* (1.5)		1000	
57	*U* (140, 220)		*U* (5, 85)		*Exp* (1.5)		50	
58	*U* (140, 220)		*U* (5, 85)		*Exp* (1.5)		100	
59	*U* (140, 220)		*U* (5, 85)		*Exp* (1.5)		150	
60	*U* (140, 220)		*U* (5, 85)		*Exp* (1.5)		200	
61	*U* (140, 220)		*U* (5, 85)		*Exp* (1.5)		300	
62	*U* (140, 220)		*U* (5, 85)		*Exp* (1.5)		500	
63	*U* (140, 220)		*U* (5, 85)		*Exp* (1.5)		1000	
64	*Exp* (180)		*Exp* (45)		*Exp* (2.5)		50	
65	*Exp* (180)		*Exp* (45)		*Exp* (2.5)		100	
66	*Exp* (180)		*Exp* (45)		*Exp* (2.5)		150	
67	*Exp* (180)		*Exp* (45)		*Exp* (2.5)		200	
68	*Exp* (180)		*Exp* (45)		*Exp* (2.5)		300	
69	*Exp* (180)		*Exp* (45)		*Exp* (2.5)		500	
70	*Exp* (180)		*Exp* (45)		*Exp* (2.5)		1000	
71	*Exp* (185)		*Exp* (50)		*Exp* (2.5)		50	
72	*Exp* (185)		*Exp* (50)		*Exp* (2.5)		100	
73	*Exp* (185)		*Exp* (50)		*Exp* (2.5)		150	
74	*Exp* (185)		*Exp* (50)		*Exp* (2.5)		200	
75	*Exp* (185)		*Exp* (50)		*Exp* (2.5)		300	
76	*Exp* (185)		*Exp* (50)		*Exp* (2.5)		500	
77	*Exp* (185)		*Exp* (50)		*Exp* (2.5)		1000	
78	*Exp* (190)		*Exp* (55)		*Exp* (2.5)		50	
79	*Exp* (190)		*Exp* (55)		*Exp* (2.5)		100	
80	*Exp* (190)		*Exp* (55)		*Exp* (2.5)		150	
81	*Exp* (190)		*Exp* (55)		*Exp* (2.5)		200	
82	*Exp* (190)		*Exp* (55)		*Exp* (2.5)		300	
83	*Exp* (190)		*Exp* (55)		*Exp* (2.5)		500	
84	*Exp* (190)		*Exp* (55)		*Exp* (2.5)		1000	

Some of technical terms and notations used in this table are defined as follows: volumetric intensity = number of discontinuity centres per rock volume; *N*(*i*, *j*^2^) = normal distribution, where *i* represents the mean and *j* the standard derivation; *Exp*(*k*) = exponential distribution, where *k* represents the mean; ln*N*(*l*, *m*^2^) = lognormal distribution, where *l* represents the location parameter and *m* the scale parameter; and *U*(*n*, *p*) = uniform distribution, where *n* represents the lower limit and *p* the upper limit.

**Table 2:** Probability distribution of orientations corrected using the proposed method.

Group	Dip direction (°)	Dip angle (°)	Group	Dip direction (°)	Dip angle (°)
1	*N* (178.3, 8.0^2^)	*N* (46.1, 8.1^2^)	43	*U* (160.3, 200.2)	*U* (25.0, 64.9)
2	*N* (178.8, 8.5^2^)	*N* (46.5, 8.9^2^)	44	*U* (160.4, 199.9)	*U* (25.0, 64.9)
3	*N* (179.3, 8.5^2^)	*N* (45.6, 9.4^2^)	45	*U* (160.3, 199.9)	*U* (25.0, 64.9)
4	*N* (179.0, 9.2^2^)	*N* (45.3, 9.0^2^)	46	*U* (160.3, 199.9)	*U* (25.0, 64.9)
5	*N* (178.9, 9.4^2^)	*N* (45.5, 9.4^2^)	47	*U* (160.1, 199.9)	*U* (25.0, 65.0)
6	*N* (179.5, 10.1^2^)	*N* (45.0, 10.1^2^)	48	*U* (160.1, 200.1)	*U* (25.0, 65.0)
7	*N* (179.7, 10.2^2^)	*N* (45.0, 10.1^2^)	49	*U* (160.0, 200.1)	*U* (25.0, 65.0)
8	*N* (176.9, 13.7^2^)	*N* (46.2, 11.6^2^)	50	*U* (154.5, 217.6)	*U* (13.0, 72.1)
9	*N* (177.6, 14.9^2^)	*N* (45.9, 11.5^2^)	51	*U* (153.5, 212.9)	*U* (11.4, 71.7)
10	*N* (178.2, 15.1^2^)	*N* (45.8, 12.7^2^)	52	*U* (151.7, 211.8)	*U* (12.9, 72.0)
11	*N* (178.6, 15.3^2^)	*N* (45.2, 13.9^2^)	53	*U* (152.2, 211.4)	*U* (12.5, 71.2)
12	*N* (178.9, 15.3^2^)	*N* (44.8, 14.1^2^)	54	*U* (150.7, 209.9)	*U* (13.6, 73.2)
13	*N* (178.8, 14.7^2^)	*N* (45.2, 14.3^2^)	55	*U* (150.8, 209.5)	*U* (14.2, 74.1)
14	*N* (179.4, 15.3^2^)	*N* (45.4, 14.7^2^)	56	*U* (150.3, 210.2)	*U* (15.2, 73.7)
15	*N* (185.2, 16.6^2^)	*N* (43.5, 15.7^2^)	57	*U* (145.9, 215.2)	*U* (2.0, 82.0)
16	*N* (184.4, 18.9^2^)	*N* (40.2, 18.2^2^)	58	*U* (141.3, 218.9)	*U* (1.4, 78.5)
17	*N* (184.0, 19.9^2^)	*N* (43.2, 19.8^2^)	59	*U* (142.4, 219.5)	*U* (2.7, 80.4)
18	*N* (182.5, 19.2^2^)	*N* (43.2, 18.9^2^)	60	*U* (142.4, 213.6)	*U* (3.0, 80.6)
19	*N* (181.9, 18.6^2^)	*N* (44.5, 18.0^2^)	61	*U* (141.3, 219.0)	*U* (3.8, 81.6)
20	*N* (180.9, 18.9^2^)	*N* (43.1, 18.4^2^)	62	*U* (142.8, 214.0)	*U* (5.7, 82.7)
21	*N* (180.2, 19.8^2^)	*N* (44.5, 18.9^2^)	63	*U* (140.1, 218.8)	*U* (6.2, 84.1)
22	ln*N* (5.203, 0.038^2^)	ln*N* (3.819, 0.166^2^)	64	*Exp* (355.5)	*Exp* (87.4)
23	ln*N* (5.198, 0.050^2^)	ln*N* (3.803, 0.197^2^)	65	*Exp* (311.6)	*Exp* (83.1)
24	ln*N* (5.200, 0.055^2^)	ln*N* (3.795, 0.222^2^)	66	*Exp* (107.3)	*Exp* (79.1)
25	ln*N* (5.199, 0.056^2^)	ln*N* (3.797, 0.208^2^)	67	*Exp* (133.1)	*Exp* (72.6)
26	ln*N* (5.198, 0.055^2^)	ln*N* (3.796, 0.211^2^)	68	*Exp* (102.6)	*Exp* (82.5)
27	ln*N* (5.196, 0.057^2^)	ln*N* (3.796, 0.220^2^)	69	*Exp* (104.5)	*Exp* (81.7)
28	ln*N* (5.194, 0.056^2^)	ln*N* (3.796, 0.216^2^)	70	*Exp* (109.0)	*Exp* (82.2)
29	ln*N* (5.210, 0.055^2^)	ln*N* (3.869, 0.242^2^)	71	*Exp* (81.7)	*Exp* (71.7)
30	ln*N* (5.199, 0.070^2^)	ln*N* (3.831, 0.256^2^)	72	*Exp* (104.6)	*Exp* (85.8)
31	ln*N* (5.194, 0.077^2^)	ln*N* (3.752, 0.310^2^)	73	*Exp* (118.4)	*Exp* (62.9)
32	ln*N* (5.199, 0.075^2^)	ln*N* (3.764, 0.295^2^)	74	*Exp* (110.9)	*Exp* (61.9)
33	ln*N* (5.197, 0.077^2^)	ln*N* (3.752, 0.306^2^)	75	*Exp* (102.5)	*Exp* (70.6)
34	ln*N* (5.194, 0.077^2^)	ln*N* (3.757, 0.296^2^)	76	*Exp* (99.9)	*Exp* (73.2)
35	ln*N* (5.194, 0.078^2^)	ln*N* (3.755, 0.302^2^)	77	*Exp* (99.1)	*Exp* (76.5)
36	ln*N* (5.210, 0.076^2^)	ln*N* (3.845, 0.349^2^)	78	*Exp* (116.3)	*Exp* (69.5)
37	ln*N* (5.197, 0.088^2^)	ln*N* (3.801, 0.376^2^)	79	*Exp* (141.4)	*Exp* (68.1)
38	ln*N* (5.196, 0.103^2^)	ln*N* (3.757, 0.394^2^)	80	*Exp* (114.6)	*Exp* (66.7)
39	ln*N* (5.192, 0.101^2^)	ln*N* (3.768, 0.388^2^)	81	*Exp* (107.0)	*Exp* (72.9)
40	ln*N* (5.192, 0.099^2^)	ln*N* (3.755, 0.398^2^)	82	*Exp* (117.6)	*Exp* (71.7)
41	ln*N* (5.193, 0.098^2^)	ln*N* (3.759, 0.393^2^)	83	*Exp* (117.9)	*Exp* (76.9)
42	ln*N* (5.191, 0.102^2^)	ln*N* (3.763, 0.391^2^)	84	*Exp* (118.7)	*Exp* (77.4)

**Table 3:** Volumetric intensity, diameter, aperture and size of simulated zone.

Volumetric intensity (m^−3^)	Diameter (m)	Aperture (mm)	Simulated zone		
			Length (m)	Width (m)	Height (m)
4	*Exp*(0.5)	*Exp*(1.2)	10	10	10

**Table 4:** Probability distribution of orientations corrected using the proposed method (Case II).

Sample size	Dip direction (°)	Dip angle (°)
50	*N* (276.4, 18.2^2^)	*N* (74.9, 16.9^2^)
100	*N* (277.0, 17.5^2^)	*N* (75.6, 16.0^2^)
150	*N* (279.0, 17.5^2^)	*N* (76.0, 15.6^2^)
200	*N* (278.5, 17.8^2^)	*N* (76.9, 15.2^2^)
300	*N* (277.3, 18.0^2^)	*N* (76.8, 15.2^2^)
500	*N* (277.3, 17.8^2^)	*N* (76.7, 15.3^2^)
1,000	*N* (277.6, 18.0^2^)	*N* (76.0, 15.3^2^)
