# Supplementary material for: A novel method for correcting scanline-observational bias of discontinuity orientation
Source: Sci Rep. 2016 Mar 10;6:22942. doi: 10.1038/srep22942 (PMC4785530; doi:10.1038/srep22942)
Supplement: Supplementary Information [file srep22942-s1.pdf]

Supplementary information for

A novel method for correcting scanline-observational bias of discontinuity orientation

Lei Huang, Huiming Tang, Qinwen Tan, Dingjian Wang, Liangqing Wang, Mutasim A. M. Ez Eldin, Changdong Li & Qiong Wu

Supplementary Figures

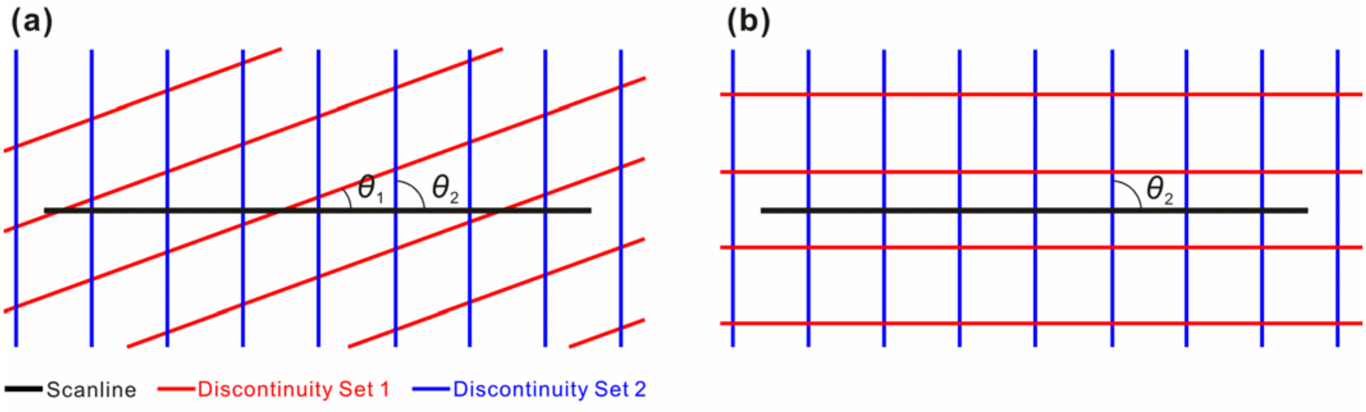

Supplementary Figure S1 Generalized two-dimensional model showing the intersection between the scanline and discontinuity sets for (a) $\theta_1 < \theta_2$; (b) extreme case $\theta_1 = 0\ ° < \theta_2$

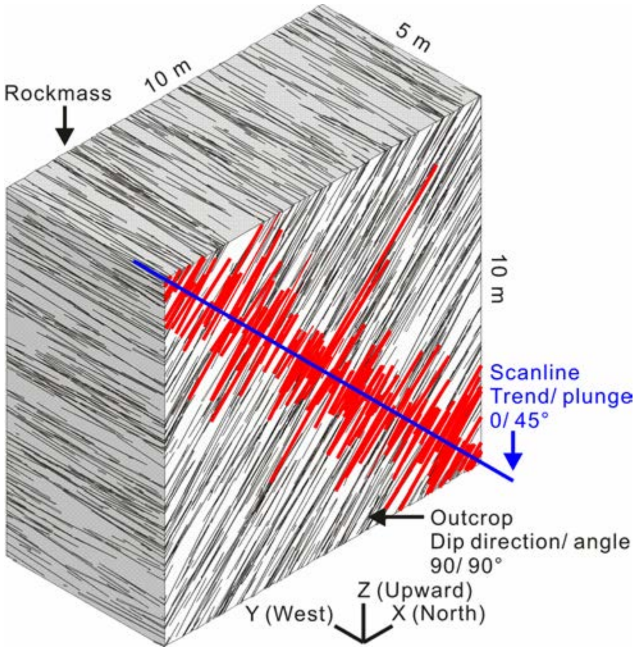

Supplementary Figure S2 Discrete fracture network model with the uniformly distributed orientation for Groups 43-49 in Table 1 in the main text. The area shaded in red indicates the discontinuities observed by the scanline

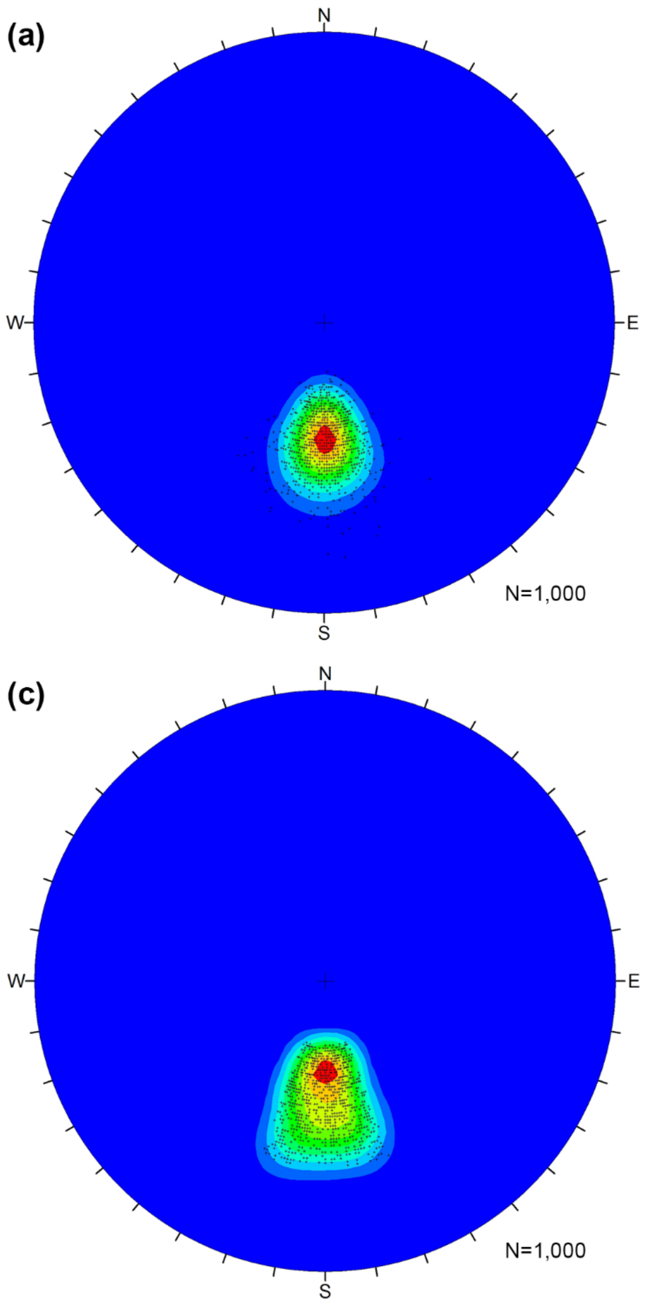

Supplementary Figure S3 Contoured stereonets of the observed orientations from (a) Group 7, (b) Group 28, (c) Group 49 and (d) Group 70. N denotes the sample size. This diagram was plotted by setting an equal angle projection of the upper hemisphere[1,2,3]. The same settings are employed in successive contoured stereonets

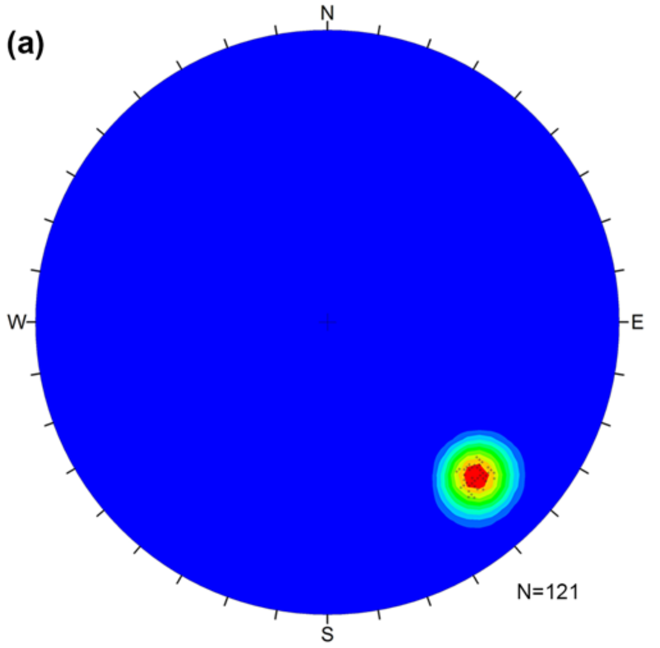

Supplementary Figure S4 Contoured stereonets of the orientations of the bedding planes (Case I). (a) Observed. (b) Corrected using the Fouché method

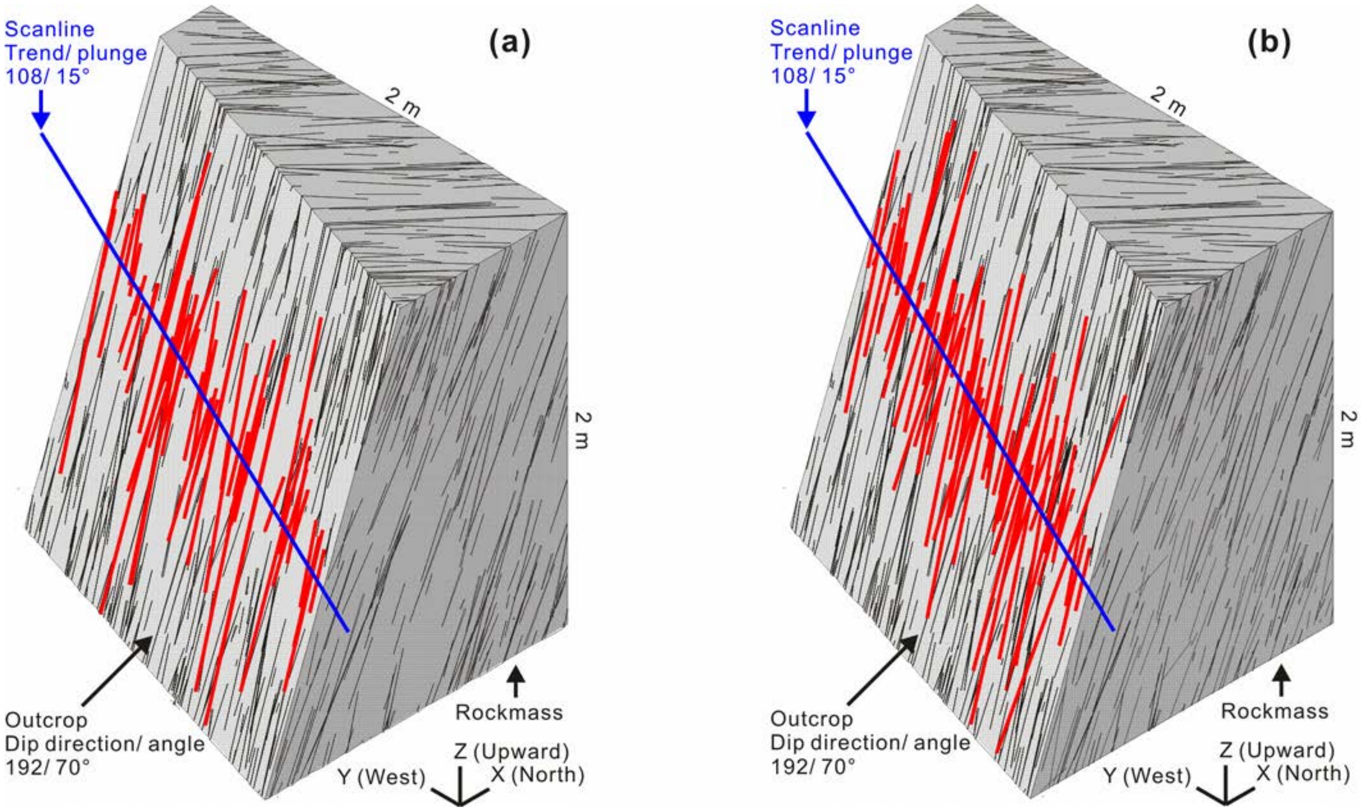

Supplementary Figure S5 Discrete fracture network models built using AutoCAD software from the corrected results of (a) the Fouché method and (b) the proposed method. The areas shaded in red indicate the discontinuities observed by the scanline

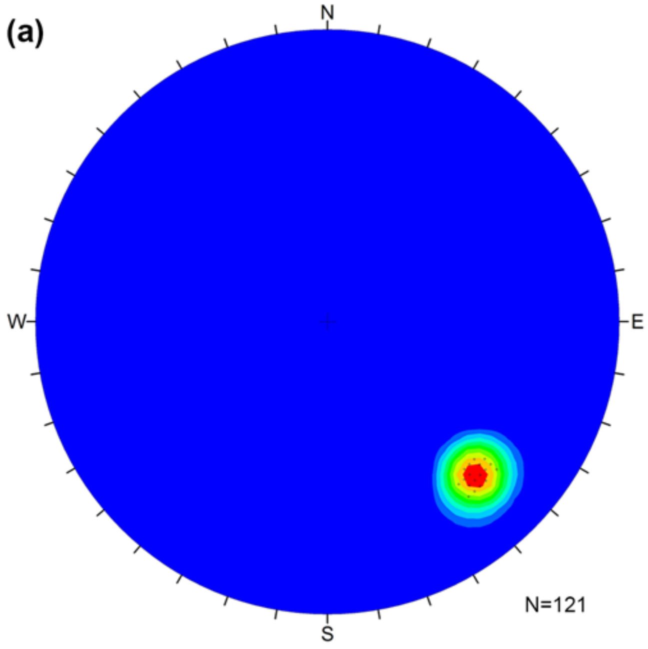

Supplementary Figure S6 Contoured stereonets of “modelled” orientations derived from the corrected results of (a) the Fouché method and (b) the proposed method

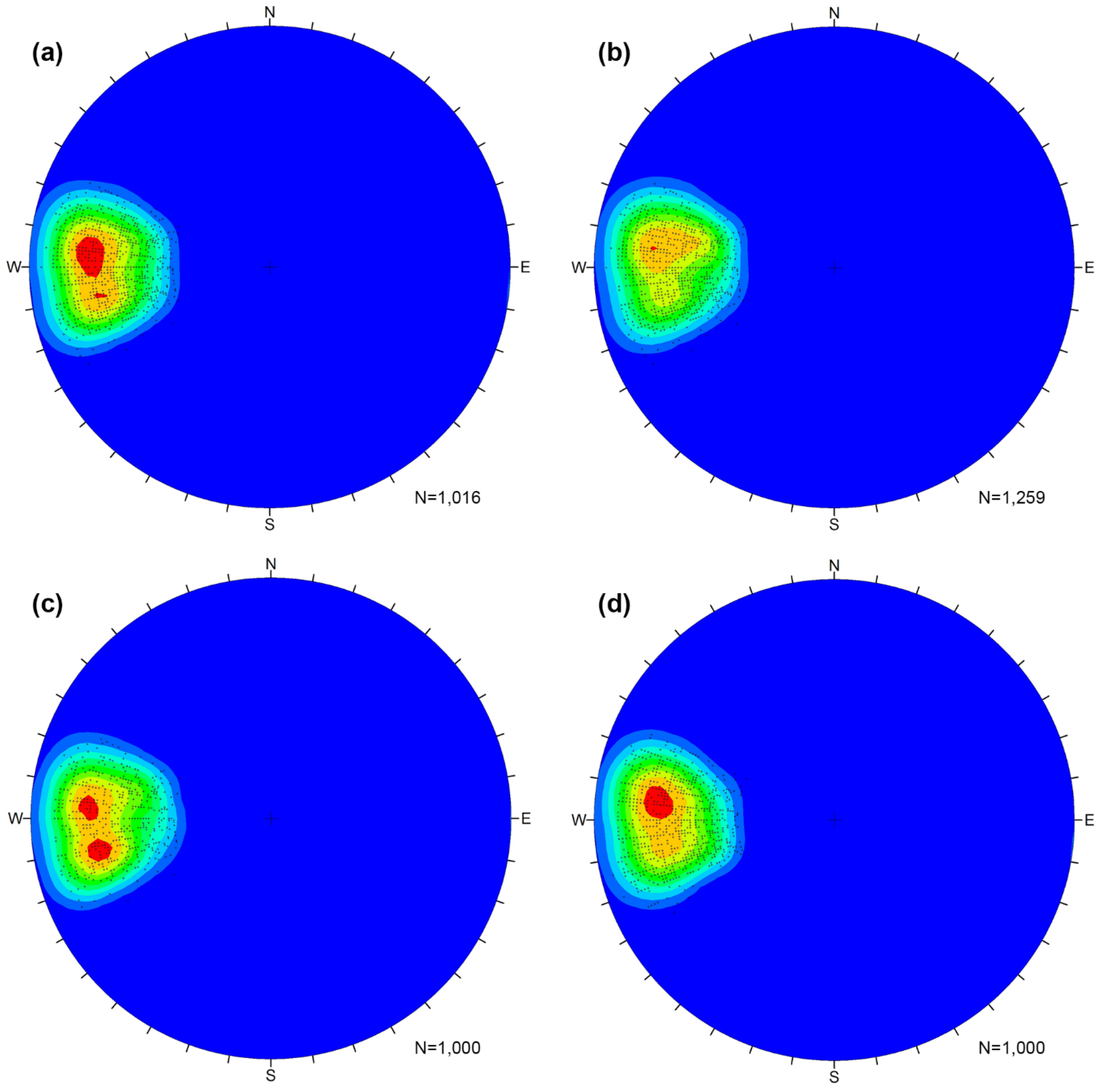

Supplementary Figure S7 Contoured stereonets of joint orientations. (a) Observed. (b) Corrected using the Fouché method from a sample of the first 1,000 observed orientations. The orientation distributions corrected by the proposed method are listed in Table 3 in the main text in terms of the probability density function. (c) “Modelled” orientations derived from the corrected results using the Fouché method. (d) “Modelled” orientations derived from the corrected results using the proposed method

Supplementary Tables

Supplementary Table S1 Two-tailed significance returned by Pearson's chi-square test for determining the independence of the dip direction and dip angle

Group	Significance	Group	Significance	Group	Significance	Group	Significance
1	0.528	22	0.756	43	0.512	64	0.992
2	0.526	23	0.344	44	0.987	65	0.907
3	0.497	24	0.483	45	0.835	66	0.704
4	0.259	25	0.301	46	0.926	67	0.874
5	0.636	26	0.912	47	0.562	68	0.838
6	0.773	27	0.568	48	0.789	69	0.483
7	0.830	28	0.089	49	0.733	70	0.209
8	0.297	29	0.492	50	0.954	71	0.429
9	0.327	30	0.827	51	0.486	72	0.863
10	0.316	31	0.874	52	0.518	73	0.537
11	0.252	32	0.629	53	0.487	74	0.292
12	0.593	33	0.810	54	0.981	75	0.415
13	0.505	34	0.754	55	0.702	76	0.217
14	0.524	35	0.424	56	0.433	77	0.246
15	0.819	36	0.396	57	0.452	78	0.942
16	0.330	37	0.607	58	0.475	79	0.859
17	0.592	38	0.256	59	0.778	80	0.803
18	0.460	39	0.316	60	0.565	81	0.925
19	0.673	40	0.313	61	0.472	82	0.478
20	0.550	41	0.372	62	0.969	83	0.938
21	0.543	42	0.552	63	0.987	84	0.260

Supplementary Appendix A: Derivation of the Terzaghi equation

The Terzaghi equation was given in a fairly concise mathematical derivation in the 1960s[4]. A more detailed derivation is provided here using analytic geometry, probability theory and integrals.

Let Set *A* represent the discontinuities that exist in a rockmass and Set *B* the subset of those discontinuities intersected by a scanline. Then, the conditional probability density $p_{B|A}(\alpha,\beta)$ denotes the likelihood Set *B* occurs, given that Set *A* is certain. Here, the case that Set *A* is certain means the dip direction and dip angle of all discontinuities in the rockmass are uniform. In other words, all the discontinuities in the rockmass are parallel (Supplementary Fig. S8). Hence, $p_{B|A}(\alpha,\beta)$ represents the probability density of the discontinuities observed by the scanline given that all of the discontinuities are parallel.

For these parallel discontinuities as shown in Supplementary Fig. S8, the scanline length, *l*, is

$$l = \frac{L}{\sin\theta} \tag{1}$$

then

$$p_{B|A}(\alpha,\beta) \propto \frac{N}{l} = \frac{N\sin\theta}{L} \tag{2}$$

Equation (2) is equivalent to

$$p_{B|A}(\alpha,\beta) = k\sin\theta\ ,\quad \alpha\in[\alpha_1,\alpha_N]\ ,\quad \beta\in[\beta_1,\beta_N] \tag{3}$$

where k is the undetermined coefficient.

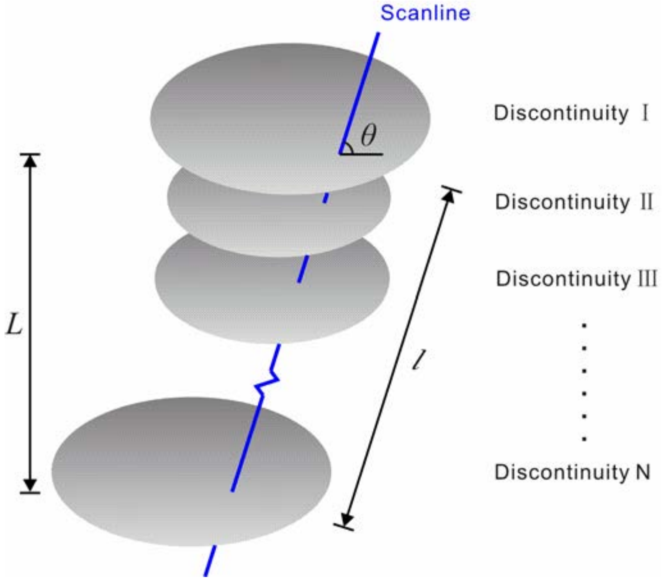

Supplementary Figure S8 Intersection between the scanline and the parallel discontinuities at angle θ. The discontinuities are parallel. The distance between Discontinuity 1 and Discontinuity N is L

The joint probability density of the dip direction and dip angle occurring in rockmass, $p_A(\alpha,\beta)$, is

$$p_A(\alpha,\beta) = \frac{p_{AB}(\alpha,\beta)}{p_{B|A}(\alpha,\beta)} \tag{4}$$

where $p_{AB}(\alpha,\beta)$ is the joint probability density of the dip direction and dip angle observed by the scanline.

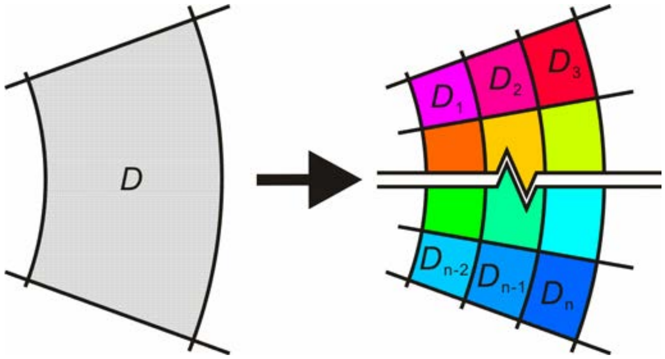

Supplementary Figure S9 Subdivision of grid

Any grid can be divided into n sub-grids (Supplementary Fig. S9). Thus, the probability of the given orientation lying in

this grid can be regarded as the sum of the probabilities lying in these sub-grids

$$\iint_D p_A(\alpha,\beta)d\alpha d\beta = \sum_{i=1}^{n}\iint_{\sigma_i} p_A(\alpha,\beta)d\alpha d\beta \tag{5}$$

Substituting Equation (3) into (4) and then substituting (4) into (5), we obtain

$$\iint_D p_A(\alpha,\beta)d\alpha d\beta = \frac{1}{k}\sum_{i=1}^{n}\iint_{\sigma_i} \frac{p_{AB}(\alpha,\beta)}{\sin\theta} d\alpha d\beta \tag{6}$$

The sinθ in Equation (6) is a function of the variables α and β. Suppose θ_{ci} is the intersection angle between the scanline and the discontinuity mapped at the sub-grid centre. Only if the sub-grid is infinitesimal (in other words, $n\to\infty$), is the variable sinθ equal to the constant sinθ_{ci}. In this case only, Equation (6) can be rewritten as

$$\iint_D p_A(\alpha,\beta)d\alpha d\beta = \frac{1}{k}\lim_{n\to\infty}\sum_{i=1}^{n}\frac{\iint_{\sigma_i} p_{AB}(\alpha,\beta)d\alpha d\beta}{\sin\theta_{ci}} = \frac{1}{k}\lim_{n\to\infty}\sum_{i=1}^{n}\frac{P_i}{\sin\theta_{ci}} \tag{7}$$

where P_i is the probability of the observed orientations within the sub-grid D_i.

However, a real sub-grid cannot be infinitesimal. In this case, the above substitution means that all nonparallel discontinuities lying in the sub-grid D_i are compulsively approximated as parallel discontinuities with a uniform orientation mapped at the sub-grid centre (Supplementary Fig. S10). This compulsive substitution leads to the error under consideration as expressed below:

$$\iint_D p_A(\alpha,\beta)d\alpha d\beta \approx \frac{1}{k}\sum_{i=1}^{n}\frac{P_i}{\sin\theta_{ci}} \tag{8}$$

Equation (8) is a Terzaghi equation[4].

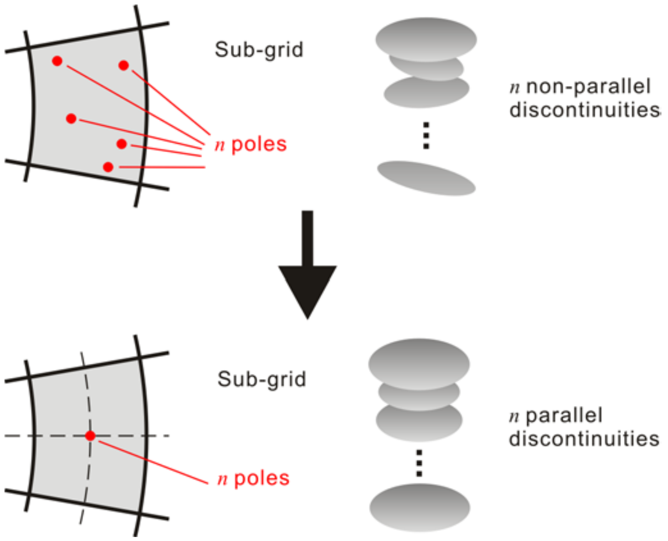

Supplementary Figure S10 Interpretation of the approximate substitution in the Terzaghi equation

Supplementary Appendix B: Conversions for avoiding the statistical break of dip direction

The method proposed here uses the dip direction/dip angle to delineate orientation. This linear delineation is subjected to the limitation that when a discontinuity cluster crosses the 0 º dip direction (Supplementary Fig. S11 (a)), the statistics will break down at this degree (Supplementary Fig. S11 (b)). One of the two following conversions can be used to address this issue. The first converts the dip directions oriented to the northwest into a special form close to 0 º by subtracting 360 º:

$$a_c = a_r - 360° \tag{9}$$

where α_r and α_c are the raw and the converted dip directions, respectively. Thus, the statistics become continuous as illustrated in Supplementary Fig. S11 (c). A second alternative is to add 360 º to the dip directions oriented to the northeast:

$$a_c = a_r + 360° \tag{10}$$

By using Equation (10), the statistic break can also be avoided as illustrated in Supplementary Fig. S11 (d). From a geometrical point of view, subtracting or adding an arbitrary multiple of 360 º does not alter the dip direction, meaning the two conversions presented here maintain the dip direction.

A similar limitation may appear in discontinuity clusters close to a 0 º dip angle as seen in the following three cases of dip direction domain:

Case I when the dip direction domain is [0, 360 º], i.e. the dip direction varies over the omnibearing total direction, the statistic break is probably inevitable at the 0 º dip direction as shown in Supplementary Fig. S12.

Case II when the dip direction domain is [α_1, α_2] where α_1>0 º and α_2<360 º, i.e. the dip direction domain does not cross 0 º, the statistics are continuous as shown in Supplementary Fig. S13.

Case III when the dip direction domain is [α_1, α_2]∪[α_3, α_4] where α_3 =0 º, α_2 =360 º and $\alpha_4 < \alpha_1$ <360 º, i.e. the dip direction domain crosses 0 º but does not vary over the omnibearing total direction, the statistic break can be addressed using Equation (9) or (10) as shown in Supplementary Fig. S14.

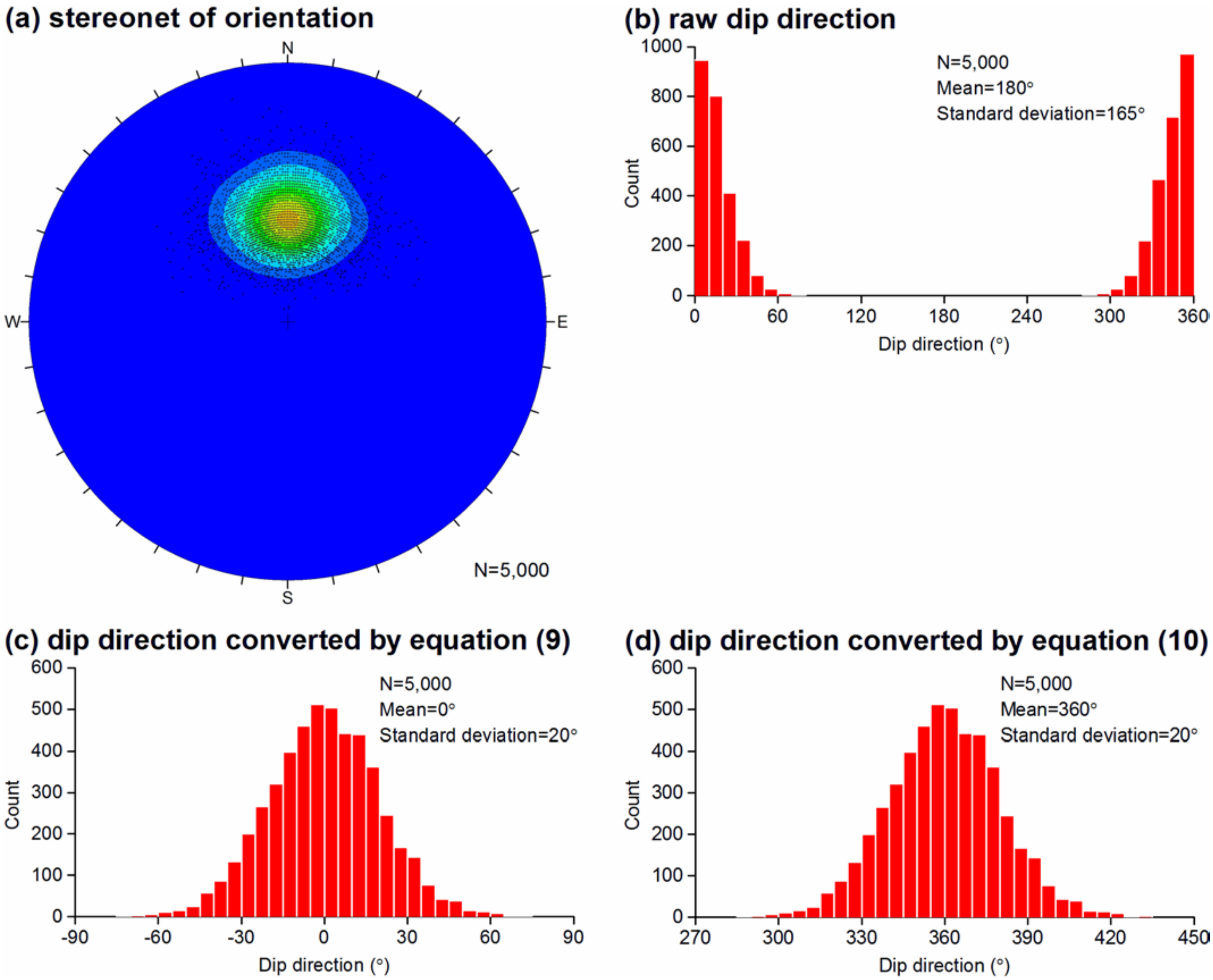

Supplementary Figure S11 The raw orientations across a 0 º dip direction and the converted ones. (a) Stereonet of orientation. (b) The raw probability distribution of the dip direction. (c) The probability distribution of the dip direction converted using Equation (9). (d) The probability distribution of the dip direction converted using Equation (10)

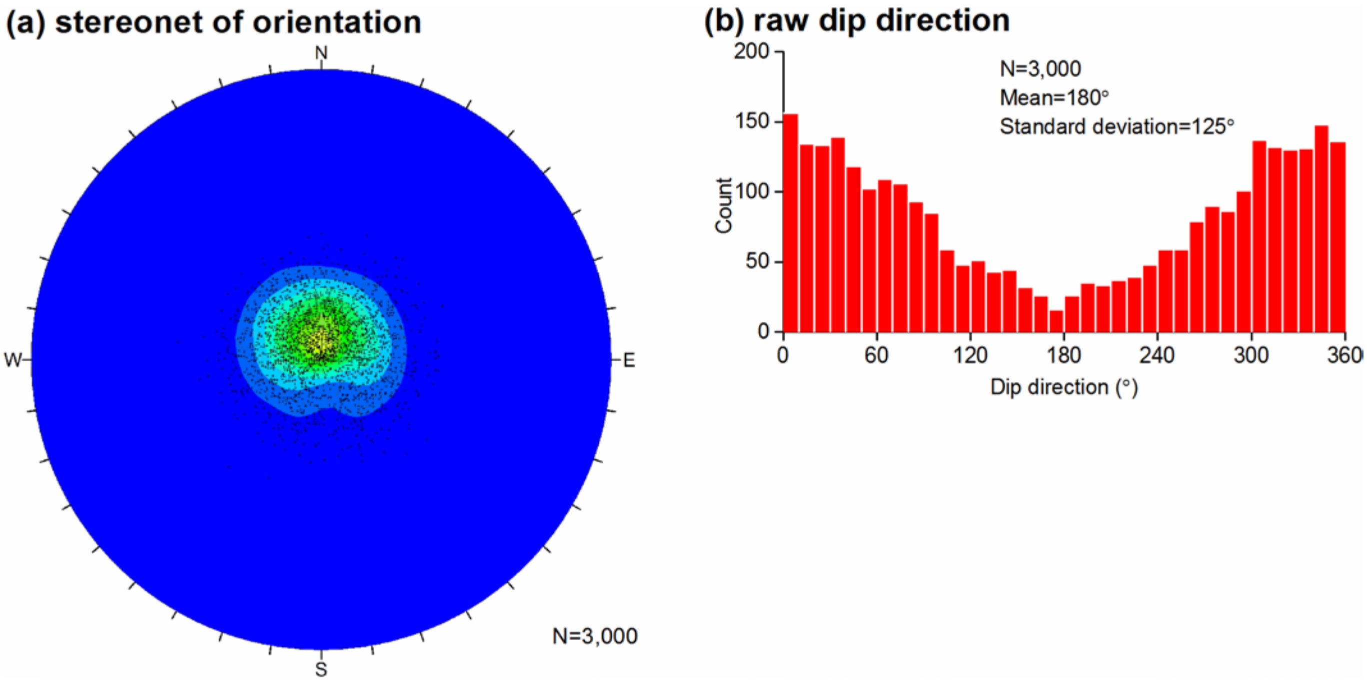

Supplementary Figure S12 The raw orientations for Case I close to a 0 ° dip angle. (a) Stereonet of orientation. (b) The raw probability distribution of the dip direction

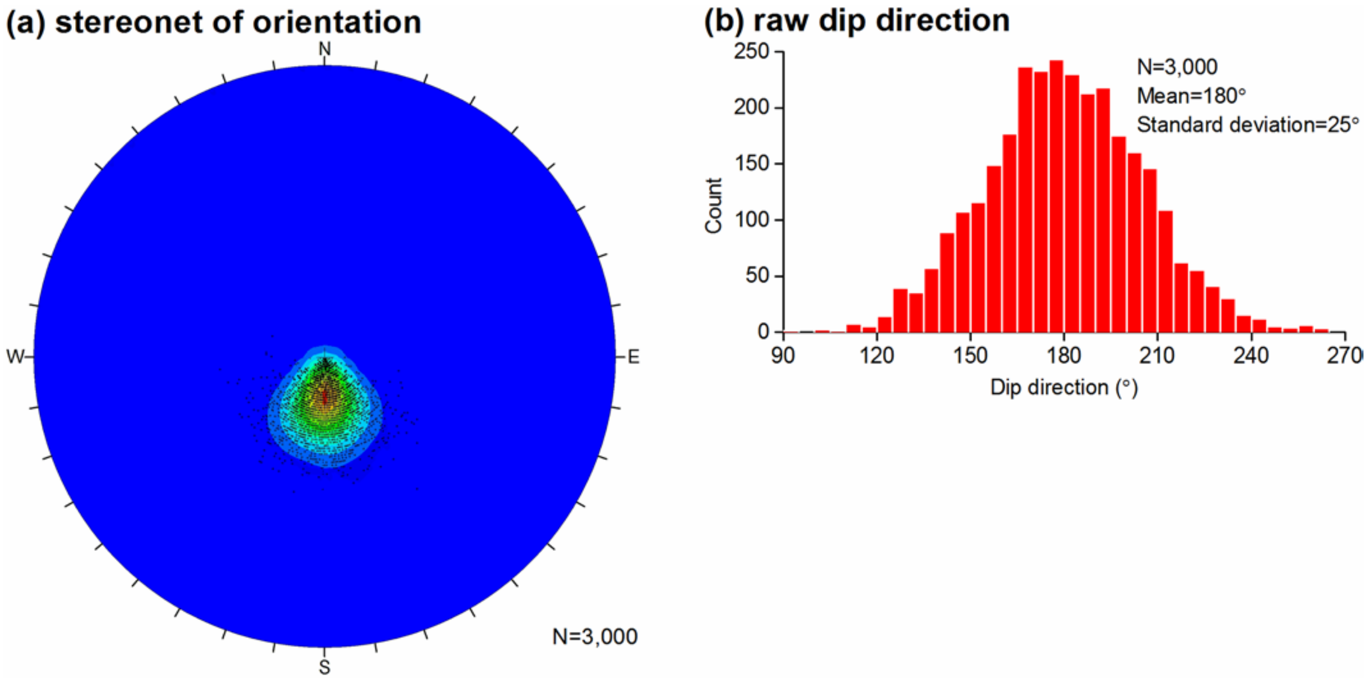

Supplementary Figure S13 The raw orientations for Case II close to a 0 ° dip angle. (a) Stereonet of orientation. (b) The raw probability distribution of the dip direction

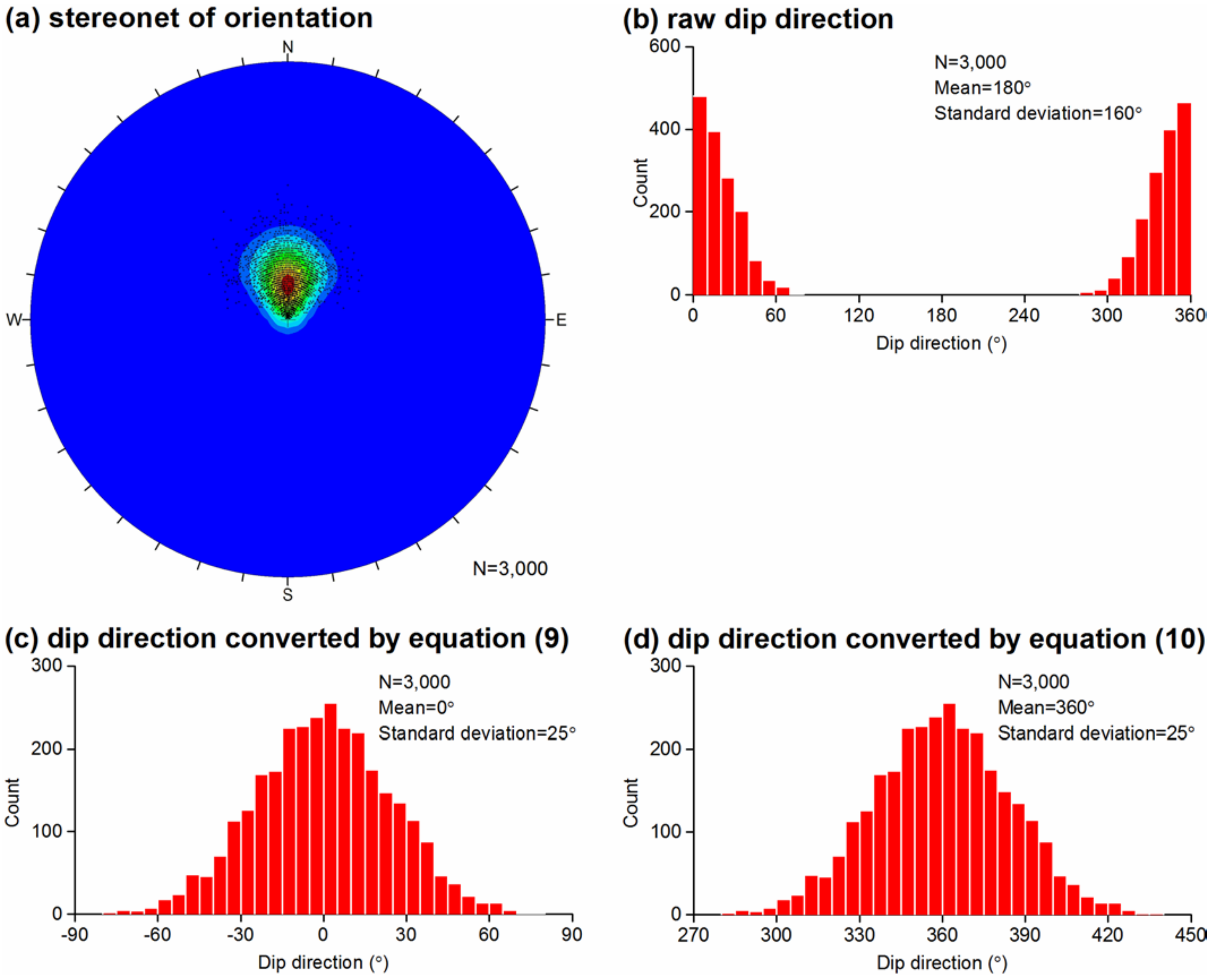

Supplementary Figure S14 The raw orientations for Case III close to a 0 ° dip angle and the converted ones. (a) Stereonet of orientation. (b) The raw probability distribution of the dip direction. (c) The probability distribution of the dip direction converted using Equation (9). (d) The probability distribution of the dip direction converted using Equation (10)

Supplementary Appendix C: Solution for the probability distribution of orientation in three-dimensional space

Recently, much research has focused on obtaining a more accurate distribution of orientation[5,6]. However, despite this effort, a solution for the probability distribution of orientation in three-dimensional space has yet to be found. This appendix derives a solution that can express the functional relationship between the distribution observed by a scanline (in one-dimensional space)

and the inherent distribution (in three-dimensional space).

For the discontinuities that exist in the three-dimensional space of a rockmass, the distribution of their dip directions and angles in terms of the joint probability density function is given:

$$p_A(\alpha,\beta) = \frac{p_{AB}(\alpha,\beta)}{k\left|\sin\beta\cos\zeta\cos(\alpha-\psi)-\cos\beta\sin\zeta\right|} \tag{11}$$

where α and β are dip direction and dip angle of discontinuity, respectively, Ψ and ζ are the trend and the plunge of the scanline, respectively; $p_A(\alpha, \beta)$ is the joint probability density function of the dip direction and dip angle in the three-dimensional space of a rockmass; $p_{AB}(\alpha, \beta)$ is the joint probability density function of the dip direction and dip angle observed by the scanline; and k is the undetermined coefficient.

The probability density function of the dip direction in three-dimensional space can be calculated from the integral of the joint probability density function determined using Equation (11) for the entire dip angle interval:

$$p_A(\alpha) = \int_{\beta\min}^{\beta_{\max}} p_A(\alpha,\beta)d\beta = \int_{\beta\min}^{\beta_{\max}} \frac{p_{AB}(\alpha,\beta)}{k\left|\sin\beta\cos\zeta\cos(\alpha-\psi)-\cos\beta\sin\zeta\right|}d\beta \tag{12}$$

where $p_A(\alpha)$ is the probability density function of the dip direction in three-dimensional space and $\beta_{\min}$ and $\beta_{\max}$ are the minimum and the maximum dip angles observed by the scanline, respectively.

Assuming that the dip direction and angle observed by the scanline are independent of each other, $p_{AB}(\alpha, \beta)$ can be expressed as the product of the probability densities of the dip direction and the dip angle:

$$p_{AB}(\alpha,\beta) = p_{AB}(\alpha)\bullet p_{AB}(\beta) \tag{13}$$

where $p_{AB}(\alpha)$ and $p_{AB}(\beta)$ are the probability density functions of the dip direction and the dip angle observed by the scanline, respectively.

Substituting Equation (13) into (12), we obtain

$$p_A(\alpha) = \frac{p_{AB}(\alpha)}{k}\int_{\beta\min}^{\beta_{\max}} \frac{p_{AB}(\beta)}{\left|\sin\beta\cos\zeta\cos(\alpha-\psi)-\cos\beta\sin\zeta\right|}d\beta \tag{14}$$

where k is to be solved as follows.

The cumulative probability of any variable in the entire domain is 1 as formulated:

$$\int_{\alpha\min}^{\alpha_{\max}} p_A(\alpha)d\alpha = 1 \tag{15}$$

where $\alpha_{\min}$ and α_{max} are the minimum and the maximum dip directions observed by the scanline, respectively.

Substituting Equation (14) into (15), k is solved as

$$k=\int_{\alpha_{\min}}^{\alpha_{\max}} p_{AB}(\alpha)d\alpha\int_{\beta_{\min}}^{\beta_{\max}}\frac{p_{AB}(\beta)}{\left|\sin\beta\cos\zeta\cos(\alpha-\psi)-\cos\beta\sin\zeta\right|}d\beta \tag{16}$$

Then, substituting Equation (16) into (14), the probability density function of the dip direction in three-dimensional space of a rockmass is determined as

$$p_A(\alpha)=\frac{p_{AB}(\alpha)\int_{\beta_{\min}}^{\beta_{\max}}\frac{p_{AB}(\beta)}{\left|\sin\beta\cos\zeta\cos(\alpha-\psi)-\cos\beta\sin\zeta\right|}d\beta}{\int_{\alpha_{\min}}^{\alpha_{\max}} p_{AB}(\alpha)d\alpha\int_{\beta_{\min}}^{\beta_{\max}}\frac{p_{AB}(\beta)}{\left|\sin\beta\cos\zeta\cos(\alpha-\psi)-\cos\beta\sin\zeta\right|}d\beta} \tag{17}$$

The cumulative distribution function of the dip direction in three-dimensional space can be calculated by integrating the probability density function determined by Equation (17) as follows:

$$F_A(\alpha)=\int_{\alpha_{\min}}^{\alpha} p_A(\alpha)d\alpha=\frac{\int_{\alpha_{\min}}^{\alpha} p_{AB}(\alpha)d\alpha\int_{\beta_{\min}}^{\beta_{\max}}\frac{p_{AB}(\beta)}{\left|\sin\beta\cos\zeta\cos(\alpha-\psi)-\cos\beta\sin\zeta\right|}d\beta}{\int_{\alpha_{\min}}^{\alpha_{\max}} p_{AB}(\alpha)d\alpha\int_{\beta_{\min}}^{\beta_{\max}}\frac{p_{AB}(\beta)}{\left|\sin\beta\cos\zeta\cos(\alpha-\psi)-\cos\beta\sin\zeta\right|}d\beta} \tag{18}$$

Similar to Equations (17) and (18) for the dip direction, the probability density function of the dip angle in three-dimensional space is derived:

$$p_A(\beta)=\frac{p_{AB}(\beta)\int_{\alpha_{\min}}^{\alpha_{\max}}\frac{p_{AB}(\alpha)}{\left|\sin\beta\cos\zeta\cos(\alpha-\psi)-\cos\beta\sin\zeta\right|}d\alpha}{\int_{\beta_{\min}}^{\beta_{\max}} p_{AB}(\beta)d\beta\int_{\alpha_{\min}}^{\alpha_{\max}}\frac{p_{AB}(\alpha)}{\left|\sin\beta\cos\zeta\cos(\alpha-\psi)-\cos\beta\sin\zeta\right|}d\alpha} \tag{19}$$

and the cumulative distribution function of the dip angle in three-dimensional space is derived:

$$F_A(\beta)=\frac{\int_{\beta_{\min}}^{\beta} p_{AB}(\beta)d\beta\int_{\alpha_{\min}}^{\alpha_{\max}}\frac{p_{AB}(\alpha)}{\left|\sin\beta\cos\zeta\cos(\alpha-\psi)-\cos\beta\sin\zeta\right|}d\alpha}{\int_{\beta_{\min}}^{\beta_{\max}} p_{AB}(\beta)d\beta\int_{\alpha_{\min}}^{\alpha_{\max}}\frac{p_{AB}(\alpha)}{\left|\sin\beta\cos\zeta\cos(\alpha-\psi)-\cos\beta\sin\zeta\right|}d\alpha} \tag{20}$$

In Equation (17), the integral $\int_{\beta_{\min}}^{\beta_{\max}}\frac{p_{AB}(\beta)}{\left|\sin\beta\cos\zeta\cos(\alpha-\psi)-\cos\beta\sin\zeta\right|}d\beta$ is difficult to transform into an analytic solution. The same difficulties also occur in the following three equations: $\int_{\beta_{\min}}^{\beta_{\max}}\frac{p_{AB}(\beta)}{\left|\sin\beta\cos\zeta\cos(\alpha-\psi)-\cos\beta\sin\zeta\right|}d\beta$ in Equation (18), $\int_{\alpha_{\min}}^{\alpha_{\max}}\frac{p_{AB}(\alpha)}{\left|\sin\beta\cos\zeta\cos(\alpha-\psi)-\cos\beta\sin\zeta\right|}d\alpha$ in Equation (19) and $\int_{\alpha_{\min}}^{\alpha_{\max}}\frac{p_{AB}(\alpha)}{\left|\sin\beta\cos\zeta\cos(\alpha-\psi)-\cos\beta\sin\zeta\right|}d\alpha$ in Equation (20). To avoid these difficulties, an approximate numerical solution

is derived as follows.

It is supposed that orientations of *n* discontinuities (i.e. dip direction/ dip angle α_1/β_1, α_2/β_2, α_3/β_3, ..., and α_n/β_n) are observed by line sampling, where $\alpha_1 \le \alpha_2 \le \alpha_3 \le \cdots \le \alpha_n$ and $\beta_1 \le \beta_2 \le \beta_3 \le \cdots \le \beta_n$. According to statistics, the observed probability is uniform within all the intervals segmented by observation data, expressed more specifically as:

for the dip direction

$$P_{AB}(\alpha_j < \alpha < \alpha_{j+1}) = \int_{\alpha_j}^{\alpha_{j+1}} p_{AB}(\alpha)d\alpha = \frac{F_{AB}(\alpha_n) - F_{AB}(\alpha_1)}{n-1}, \quad j = 1, 2, 3, \cdots, n-1 \tag{21}$$

for the dip angle

$$P_{AB}(\beta_k < \beta < \beta_{k+1}) = \int_{\beta_k}^{\beta_{k+1}} p_{AB}(\beta)d\beta = \frac{F_{AB}(\beta_n) - F_{AB}(\beta_1)}{n-1}, \quad k = 1, 2, 3, \cdots, n-1 \tag{22}$$

where $P_{AB}(\alpha_j < \alpha < \alpha_{j+1})$ is the probability of the dip direction observed by the scanline for the interval $(\alpha_j, \alpha_{j+1}]$ and $P_{AB}(\beta_k < \beta < \beta_{k+1})$ is the probability of the dip angle observed by the scanline for the interval $(\beta_k, \beta_{k+1}]$.

Then, the integrand in Equation (21) can be approximated as

$$p_{AB}(\alpha) \approx \frac{F_{AB}(\alpha_n) - F_{AB}(\alpha_1)}{(n-1)(\alpha_{j+1} - \alpha_j)}, \quad \alpha \in (\alpha_j, \alpha_{j+1}) \tag{23}$$

Similarly, the integrand in Equation (22) is approximated as

$$p_{AB}(\beta) \approx \frac{F_{AB}(\beta_n) - F_{AB}(\beta_1)}{(n-1)(\beta_{k+1} - \beta_k)}, \quad \beta \in (\beta_k, \beta_{k+1}) \tag{24}$$

Then, Equation (24) is substituted for an integral in Equation (17):

$$\begin{aligned}\int_{\beta_{\min}}^{\beta_{\max}} \frac{p_{AB}(\beta)}{\left|\sin\beta\cos\zeta\cos(\alpha-\psi) - \cos\beta\sin\zeta\right|}d\beta &\approx \sum_{k=1}^{n-1}\left[\frac{F_{AB}(\beta_n) - F_{AB}(\beta_1)}{(n-1)(\beta_{k+1} - \beta_k)}\int_{\beta_k}^{\beta_{k+1}} \frac{1}{\left|\sin\beta\cos\zeta\cos(\alpha-\psi) - \cos\beta\sin\zeta\right|}d\beta\right] \\ &= \frac{F_{AB}(\beta_n) - F_{AB}(\beta_1)}{n-1}\sum_{k=1}^{n-1}\left[\frac{1}{(\beta_{k+1} - \beta_k)}\int_{\beta_k}^{\beta_{k+1}} \frac{1}{\left|\sin\beta\cos\zeta\cos(\alpha-\psi) - \cos\beta\sin\zeta\right|}d\beta\right]\end{aligned} \tag{25}$$

It is difficult to solve the integral in Equation (25). In order to obtain an integral form, two transforming approaches (Approach 1 and Approach 2) are presented here.

Approach 1

Equation (25) is transformed into

$$\int_{\beta_{\min}}^{\beta_{\max}} \frac{p_{AB}(\beta)}{\left|\sin\beta\cos\zeta\cos(\alpha-\psi)-\cos\beta\sin\zeta\right|}d\beta = \frac{F_{AB}(\beta_n)-F_{AB}(\beta_1)}{n-1}\sum_{k=1}^{n-1}\left[\frac{1}{(\beta_{k+1}-\beta_k)}\int_{\beta_k}^{\beta_{k+1}}\frac{1}{\sqrt{\cos^2\zeta\cos^2(\alpha-\psi)+\sin^2\zeta}\left|\sin(\beta-\phi)\right|}d\beta\right]$$
$$= \frac{F_{AB}(\beta_n)-F_{AB}(\beta_1)}{(n-1)\sqrt{\cos^2\zeta\cos^2(\alpha-\psi)+\sin^2\zeta}}\sum_{k=1}^{n-1}\left[\frac{1}{(\beta_{k+1}-\beta_k)}\int_{\beta_k}^{\beta_{k+1}}\frac{1}{\left|\sin(\beta-\phi)\right|}d\beta\right] \tag{26}$$

with

$$\phi = \arctan\frac{\sin\zeta}{\cos\zeta\cos(\alpha-\psi)} \tag{27}$$

The definite integral in Equation (26) is transformed into

$$\int_{\beta_k}^{\beta_{k+1}}\frac{1}{\left|\sin(\beta-\phi)\right|}d\beta = \begin{cases}\left[\ln\left|\csc(\beta-\phi)-\cot(\beta-\phi)\right|\right]_{\beta_k}^{\beta_{k+1}}, \text{ if } \sin(\beta-\phi)>0 \\ -\left[\ln\left|\csc(\beta-\phi)-\cot(\beta-\phi)\right|\right]_{\beta_k}^{\beta_{k+1}}, \text{ if } \sin(\beta-\phi)<0\end{cases} \tag{28}$$

Equation (28) is substituted into (26), which is then substituted into (17); after the substitution, the judgment criteria $\sin(\beta-\phi)>0$ and the solution are both complicated. To find a more valid solution, a second approach is tried.

Approach 2

Equation (25) is rewritten as

$$\int_{\beta_{\min}}^{\beta_{\max}} \frac{p_{AB}(\beta)}{\left|\sin\beta\cos\zeta\cos(\alpha-\psi)-\cos\beta\sin\zeta\right|}d\beta \approx \sum_{k=1}^{n-1}\left[\frac{F_{AB}(\beta_n)-F_{AB}(\beta_1)}{(n-1)(\beta_{k+1}-\beta_k)}\int_{\beta_k}^{\beta_{k+1}}\frac{1}{\left|\sin\beta\cos\zeta\cos(\alpha-\psi)-\cos\beta\sin\zeta\right|}d\beta\right]$$
$$= \frac{F_{AB}(\beta_n)-F_{AB}(\beta_1)}{n-1}\sum_{k=1}^{n-1}\left[\frac{1}{\beta_{k+1}-\beta_k}\int_{\beta_k}^{\beta_{k+1}}\frac{1}{\left|\sin\beta\cos\zeta\cos(\alpha-\psi)-\cos\beta\sin\zeta\right|}d\beta\right]$$
$$\approx \frac{F_{AB}(\beta_n)-F_{AB}(\beta_1)}{n-1}\sum_{k=1}^{n-1}\left[\frac{1}{\beta_{k+1}-\beta_k}\bullet\frac{\beta_{k+1}-\beta_k}{\left|\sin\frac{\beta_k+\beta_{k+1}}{2}\cos\zeta\cos(\alpha-\psi)-\cos\frac{\beta_k+\beta_{k+1}}{2}\sin\zeta\right|}\right]$$
$$= \frac{F_{AB}(\beta_n)-F_{AB}(\beta_1)}{n-1}\sum_{k=1}^{n-1}\frac{1}{\left|\sin\frac{\beta_k+\beta_{k+1}}{2}\cos\zeta\cos(\alpha-\psi)-\cos\frac{\beta_k+\beta_{k+1}}{2}\sin\zeta\right|}$$
$$k=1,2,3,\cdots,n-1 \tag{29}$$

Compared with the first approach, Approach 2 using Equation (29) is simpler. Thus, Approach 2 is adopted to transform the integral in Equation (25).

Equations (23) and (29) are substituted for the numerator in (17)

$$p_{AB}(\alpha)\int_{\beta_{\min}}^{\beta_{\max}} \frac{p_{AB}(\beta)}{\left|\sin\beta\cos\zeta\cos(\alpha-\psi)-\cos\beta\sin\zeta\right|}d\beta$$

$$=\frac{[F_{AB}(\alpha_n)-F_{AB}(\alpha_1)][(F_{AB}(\beta_n)-F_{AB}(\beta_1)]}{(\alpha_{j+1}-\alpha_j)(n-1)^2}\sum_{k=1}^{n-1}\frac{1}{\left|\sin\frac{\beta_k+\beta_{k+1}}{2}\cos\zeta\cos(\alpha-\xi)-\cos\frac{\beta_k+\beta_{k+1}}{2}\sin\zeta\right|}$$

$$\approx\frac{[F_{AB}(\alpha_n)-F_{AB}(\alpha_1)][(F_{AB}(\beta_n)-F_{AB}(\beta_1)]}{(\alpha_{j+1}-\alpha_j)(n-1)^2}\sum_{k=1}^{n-1}\frac{1}{\left|\sin\frac{\beta_k+\beta_{k+1}}{2}\cos\zeta\cos(\frac{\alpha_j+\alpha_{j+1}}{2}-\xi)-\cos\frac{\beta_k+\beta_{k+1}}{2}\sin\zeta\right|}$$

$$j=1,2,3,\cdots,n-1;\quad \alpha\in(\alpha_j,\alpha_{j+1}] \tag{30}$$

Equation (30) is substituted for the denominator in (17)

$$\int_{\alpha_{\min}}^{\alpha_{\max}} p_{AB}(\alpha)d\alpha\int_{\beta_{\min}}^{\beta_{\max}} \frac{p_{AB}(\beta)}{\left|\sin\beta\cos\zeta\cos(\alpha-\psi)-\cos\beta\sin\zeta\right|}d\beta$$

$$=\sum_{r=1}^{n-1}\left\{(\alpha_{j+1}-\alpha_j)\frac{[F_{AB}(\alpha_n)-F_{AB}(\alpha_1)][(F_{AB}(\beta_n)-F_{AB}(\beta_1)]}{(\alpha_{j+1}-\alpha_j)(n-1)^2}\sum_{k=1}^{n-1}\frac{1}{\left|\sin\frac{\beta_k+\beta_{k+1}}{2}\cos\zeta\cos(\frac{\alpha_r+\alpha_{r+1}}{2}-\psi)-\cos\frac{\beta_k+\beta_{k+1}}{2}\sin\zeta\right|}\right\}$$

$$=\frac{[F_{AB}(\alpha_n)-F_{AB}(\alpha_1)][(F_{AB}(\beta_n)-F_{AB}(\beta_1)]}{(n-1)^2}\sum_{r=1}^{n-1}\sum_{k=1}^{n-1}\frac{1}{\left|\sin\frac{\beta_k+\beta_{k+1}}{2}\cos\zeta\cos(\frac{\alpha_r+\alpha_{r+1}}{2}-\psi)-\cos\frac{\beta_k+\beta_{k+1}}{2}\sin\zeta\right|}$$

$$\tag{31}$$

Substituting Equations (30) and (31) into (17), the probability density of the dip direction in three-dimensional space is rewritten in terms of a piecewise function as

$$p_A(\alpha)=\frac{1}{\alpha_{j+1}-\alpha_j}\bullet\frac{\sum_{k=1}^{n-1}\frac{1}{\left|\sin\frac{\beta_k+\beta_{k+1}}{2}\cos\zeta\cos(\frac{\alpha_j+\alpha_{j+1}}{2}-\psi)-\cos\frac{\beta_k+\beta_{k+1}}{2}\sin\zeta\right|}}{\sum_{r=1}^{n-1}\sum_{k=1}^{n-1}\frac{1}{\left|\sin\frac{\beta_k+\beta_{k+1}}{2}\cos\zeta\cos(\frac{\alpha_r+\alpha_{r+1}}{2}-\psi)-\cos\frac{\beta_k+\beta_{k+1}}{2}\sin\zeta\right|}},\quad j=1,2,3,\cdots,n-1;\quad \alpha\in(\alpha_j,\alpha_{j+1}] \tag{32}$$

Hence, the cumulative probability of the dip direction in three-dimensional space is rewritten as

$$P_A(\alpha_j)=\frac{\sum_{r=1}^{j-1}\sum_{k=1}^{n-1}\frac{1}{\left|\sin\frac{\beta_k+\beta_{k+1}}{2}\cos\zeta\cos(\frac{\alpha_r+\alpha_{r+1}}{2}-\psi)-\cos\frac{\beta_k+\beta_{k+1}}{2}\sin\zeta\right|}}{\sum_{r=1}^{n-1}\sum_{k=1}^{n-1}\frac{1}{\left|\sin\frac{\beta_k+\beta_{k+1}}{2}\cos\zeta\cos(\frac{\alpha_r+\alpha_{r+1}}{2}-\psi)-\cos\frac{\beta_k+\beta_{k+1}}{2}\sin\zeta\right|}},\quad j=2,3,\cdots,n \tag{33}$$

Similar to the dip direction, the cumulative probability of the dip angle in three-dimensional space is rewritten as

$$P_A(\beta_k) = \frac{\sum_{r=1}^{k-1}\sum_{j=1}^{n-1}\frac{1}{\left|\sin\frac{\beta_r+\beta_{r+1}}{2}\cos\zeta\cos(\frac{\alpha_j+\alpha_{j+1}}{2}-\psi)-\cos\frac{\beta_r+\beta_{r+1}}{2}\sin\zeta\right|}}{\sum_{r=1}^{n-1}\sum_{j=1}^{n-1}\frac{1}{\left|\sin\frac{\beta_r+\beta_{r+1}}{2}\cos\zeta\cos(\frac{\alpha_j+\alpha_{j+1}}{2}-\psi)-\cos\frac{\beta_r+\beta_{r+1}}{2}\sin\zeta\right|}},\quad k=2,3,\cdots,n \qquad (34)$$

Supplementary references

1 Rocsciences, *Dips*. *Technical report*. (2015) Available at: https://www.rocscience.com/rocscience/products/dips. (Accessed: 2nd November 2015).

2 Wu, J., Zhang, Z. X. & Kwok, C. Y. Stability analysis of rock blocks around a cross-harbor tunnel using the improved morphological visualization method. *Eng. Geol.* **187,** 10–31 (2015).

3 Li, Y., Wang, Q., Chen, J., Xu, L. & Song, S. K-means algorithm based on particle swarm optimization for the identification of rock discontinuity sets. *Rock Mech. Rock Eng.* **48,** 375–385 (2015).

4 Terzaghi, R. D. Sources of error in joint surveys. *Geotechnique* **15**, 287–304 (1965).

5 Fouché, O. & Diebolt, J. Describing the Geometry of 3D Fracture Systems by Correcting for Linear Sampling Bias. *Math. Geol.* **36,** 33–63 (2004).

6 Tang, H. *et al.* Identification and mitigation of error produced when using Terzaghi's bias correction. *Strength Mater.* (in the press).
